# The effectiveness and safety of a mobile application-based self-regulation intervention to support weight loss among adults living with obesity: a large-scale pragmatic randomised controlled trial

**DOI:** 10.1186/s12916-025-04519-8

**Published:** 2025-11-29

**Authors:** Paul Doody, Gina M. Wren, Sarah Mounsey, Simona Haasova, Cristina Stewart, Stella J. P. Haffner, Susan A. Jebb, Paul Aveyard

**Affiliations:** 1 Nuffield Department of Primary Care Health Sciences, Medical Sciences Division, University of Oxford, Oxford, United Kingdom; 2 Discipline of Public Health and Primary Care, Institute of Population Health, School of Medicine, Trinity College Dublin, the University of Dublin, Dublin, Ireland; 3 National Institute of Health Research Oxford and Thames Valley Applied Research Collaboration, Oxford, UK; 4 Smith School of Enterprise and the Environment, School of Geography and the Environment, University of Oxford, Oxford, UK; 5 The Faculty of Business and Economics, University of Lausanne, Lausanne, Switzerland; 6 School of Health and Wellbeing, University of Glasgow, Glasgow, Scotland; 7 National Institute of Health Research Oxford Health Biomedical Research Centre, Oxford, UK; 8 National Institute of Health Research Oxford Biomedical Research Centre, Oxford, UK

**Keywords:** Mobile application, Obesity, Randomised controlled trial, Self-regulation, Weight loss

## Abstract

**Background:**

Obesity is a leading risk factor for avoidable ill health. Effective interventions that can be delivered at scale are needed to support people living with excess weight to lose weight.

**Methods:**

We conducted a two-arm randomised controlled trial among adults aged ≥ 18 years living with obesity in the United Kingdom. Participants were recruited via online social media advertisements and allocated 1:1 to receive six months of unlimited access to a purpose-built mobile application based on self-regulation theory (i.e. daily self-weighing, action planning, and weekly reflections), or advice to lose weight. Co-primary outcomes were change in weight and the proportion of participants achieving ≥ 5% weight loss at six months. We also assessed whether the intervention had adverse effects on symptoms of disordered eating. All participants were included in the intention-to-treat analyses. Per-protocol analyses included participants that successfully completed a minimum of one weigh-in and action on at least four separate weeks and had at least one action in their toolbox. Data were analysed using linear mixed effects and analogous logistic models over the six-month period. The trial was registered on ClinicalTrials.gov: NCT05787652.

**Results:**

From 13 April to 15 May 2023, 1607 participants were randomly assigned to control (*n* = 806), or intervention (*n* = 801). Weight was reported by 632 (39.3%) participants at six months. Mean difference in weight change between groups at six months in intention-to-treat analyses was − 1.85 kg (95% CI: − 2.53, − 1.17, *p* < 0.001), and the odds of losing ≥ 5% were 2.11 (95% CI: 1.48, 3.03, *p* < 0.001). Per-protocol analyses showed participants using the app lost an additional − 2.18 kg compared to control, and the odds of losing ≥ 5% were 2.44 (95% CI: 1.67, 3.59, *p* < 0.001). The proportion of participants scoring above threshold for symptoms of disordered eating declined in intervention relative to control at six months (adjusted OR: 0.51, 95% CI: 0.29, 0.91, *p* = 0.024).

**Conclusions:**

An app with no human contact, designed to foster self-regulatory behaviours, increased weight loss and reduced symptoms of disordered eating in people living with obesity and could be safely deployed at the population level to support effective weight management.

**Supplementary Information:**

The online version contains supplementary material available at 10.1186/s12916-025-04519-8.

## Background

Obesity is a leading preventable risk factor for premature morbidity and mortality, and a major public health challenge globally, where it is estimated that over a third of adults now live with excess weight [1–5]. This calls for policy responses that address the drivers of obesity in the food system and environment, but also mobilisation of mass support for weight loss.

Behavioural support increases weight loss over unsupported efforts, but providing this at scale, commensurate with need, challenges health systems. Digitally delivered interventions have the potential to provide accessible, convenient, low-cost, and scalable treatment options [6]. A systematic review and meta-analysis of mobile applications supporting weight loss, including 11 randomised trials with 1591 participants and follow-up varying from six weeks to nine months, showed weight loss of − 1.07 kg (95% confidence interval (CI) − 1.92, − 0.21) relative to no intervention; providing evidence of the effectiveness of this method of intervention delivery in the context of weight loss interventions [7].

A common feature of mobile applications is that they support self-regulation with regular weighing, monitoring of behaviour, and encourage weight loss strategies. While there is no evidence that self-weighing alone increases weight loss [8], programmes that incorporate additional behavioural support alongside regular self-weighing have proven somewhat effective [9]. Self-regulation theory would posit that these programmes could be improved by supporting people to make specific time-bound plans about how weight loss actions would be enacted [10]. In our 2020 pilot study, participants were guided through the self-regulatory cycle where they performed daily self-weighing and were encouraged to experiment with different weight loss strategies each day, reflect weekly on the strategies they used, and continue to use those they found useful [11]. At eight weeks the intervention led to weight loss of 3.20 kg (95% CI − 4.49, − 1.92) greater than the control who solely engaged in self-weighing. However, engagement with the intervention involved in-person enrolment into the trial and regular emails with a sole researcher, which may have supported engagement over and above a truly self-supported weight-loss attempt.

Here, we adapted the self-regulation intervention from the pilot study, to be delivered via a mobile application with no in-person contact. The primary objective was to examine the effectiveness of this digitally delivered self-regulation intervention to support weight loss, compared to a control where participants were advised to lose weight. There was a concern that the daily focus on weight and related behaviours may lead to, or exacerbate the risk of, eating disorders, so a secondary objective was to assess the safety of the intervention by monitoring symptoms of disordered eating.

## Methods

### Study design

We conducted a two-arm, parallel group, individually randomised, controlled, superiority trial to assess the effectiveness of a purpose-built, digitally delivered, self-regulation mobile application (Adults Regulating Their weight Everyday with Mobile Internet Support (ARTEMIS)) to support weight loss compared to advice to lose weight among adults living with obesity. The trial took place between April and December 2023. Participation continued for 26 weeks from baseline to final follow-up. All study procedures were conducted remotely via the Research Electronic Database Capture (REDCap) web application or the purpose-built study app (intervention only). The trial was approved by the Central University Research Ethics Committee of the University of Oxford (R82050/RE001) and prospectively registered on ClinicalTrials.gov: NCT05787652.

### Recruitment

Participants living in the United Kingdom were recruited online through advertisements on social media (Google, Facebook, Twitter, and Instagram) between 13 April and 15 May 2023. Advertisements linked to the study landing page, where potential participants were presented with a participant information sheet (PIS). After reading the PIS, potential participants were provided a link to the study website where they could consent to participate. Following consent, eligibility was assessed through a brief online screening form. To be eligible potential participants had to be aged ≥ 18 years, with a body mass index (BMI) ≥ 30 kg/m^2^ (≥ 27.5 kg/m^2^ for non-European ethnicities). Complete eligibility criteria are detailed in Additional file 1: Table S1. If eligible, potential participants were invited to complete a short baseline questionnaire which captured contact details; socio-demographic characteristics; anthropometric measures (reporting of weight, accompanied by a photograph of the person, on scales, showing their weight, and self-reported height); a six-item disordered eating questionnaire (an abridged Eating Disorders Examination – Questionnaire Short form (EDE-QS)) [12, 13]; and responses to three questions regarding overall health, quality of life, and body satisfaction on 5-point Likert scales from 1 (poor) to 5 (excellent).

### Randomisation and masking

Participants were randomised 1:1 using a computerised algorithm with a simple randomisation schedule built into REDCap. The allocation sequence was unknown to researchers or participants, i.e. group allocation was concealed until randomisation was performed. It was not possible to blind participants to treatment allocation post-randomisation as the control group received no intervention; however, follow-up was completed remotely with no in-person contact. It was also not possible to blind the research team to treatment allocation post-randomisation due to the need for occasional engagement with participants via the trial-specific mailbox with queries regarding the app and/or follow-up assessment.

### Procedures

We have previously shown that self-monitoring of weight alone does not typically lead to completion of the self-regulatory cycle, i.e. comparing current state to a goal; reflecting on previous behaviours; forming an action plan to reach the goal; and performing the planned action(s) [10]. This intervention aimed to guide participants through each stage of the self-regulatory cycle on a daily basis, and was delivered in an automated, self-directed format without human involvement. It supported participants in experimenting with evidence-based weight loss strategies to find helpful ones for them and encouraged their repeated use. Participants had unlimited access to the app for the six-month duration of the intervention period. The intervention content and its development are detailed in Additional file 2 and described elsewhere [11, 14].

**Fig. 1:**
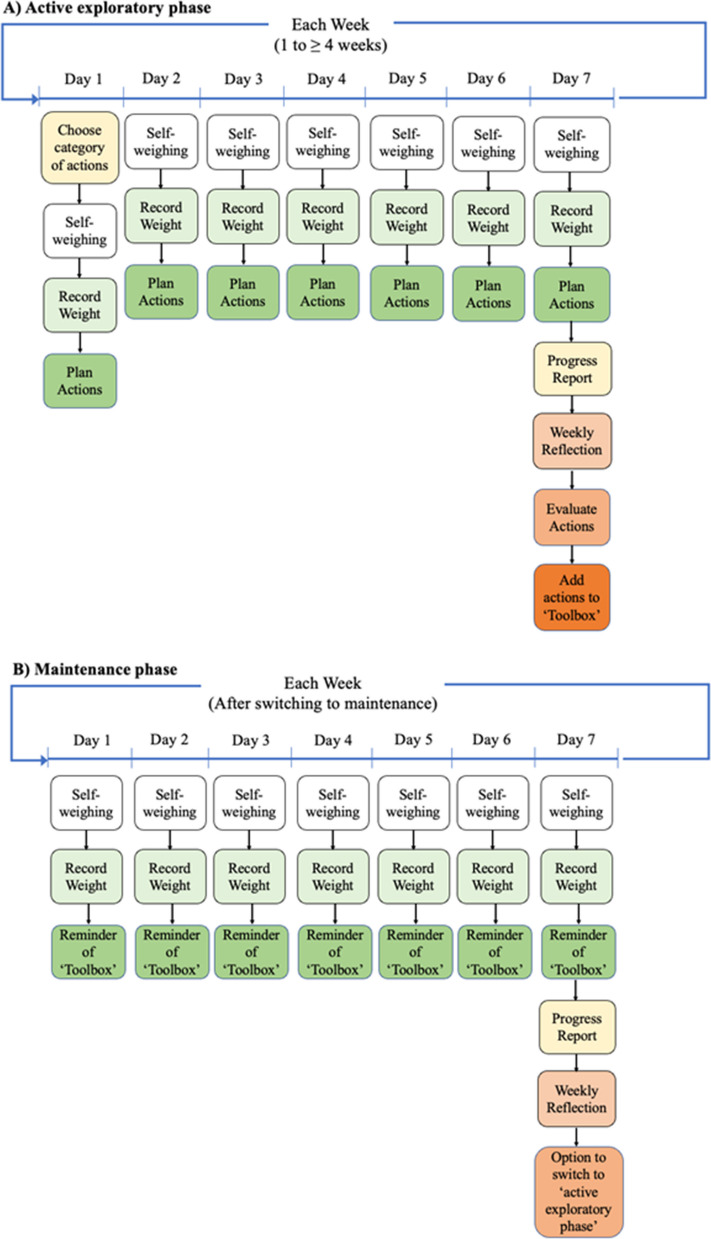
**A** Summary of intervention procedures during the ‘active exploratory phase’ (week one to at least week four) where participants could try new weight loss actions each day. **B** Summary of intervention procedures during the ‘maintenance phase’ (optional switch after week four) where participants used actions from their ‘toolbox’

Only participants randomised to the intervention arm were granted access to the ARTEMIS app, which they were instructed to download via the Apple or Google Play stores and log in using a unique one-time passcode issued at randomisation. After logging in, participants were met with two short explanatory animated subtitled videos outlining the rationale of the approach and how to use the app. Participants entered their height, weight, goal weight, preferred daily notification time, and selected a category of weight loss actions to try for the week (e.g. eating in a structured way, being more active as part of daily life). There were eight categories, relating to diet, physical activity, and sleep, which could be selected by participants and rotated weekly. Within each category were several evidence-based actions that could be chosen by participants and rotated daily (Additional file 2). Each day, participants were prompted by a push notification at a time they had selected, to weigh themselves, record their weight in the app, choose a specific weight loss action from their selected category to perform that day, and make an action plan about how, when, and where they would perform that action. Participants repeated this process every day, each time selecting a different action from their selected action category. At the end of each week, participants received a progress report which contained information on their weight change during the week, displayed on a regression line through daily weights, and their progress since beginning the intervention. Participants were asked to reflect on the usefulness of the individual strategies used that week and save those that they felt were helpful to their ‘toolbox’ (Fig. 1A).

After the fourth week, participants could choose to continue to experiment with new strategies or switch to maintenance and use the existing strategies already in their ‘*toolbox*’. In the maintenance phase, participants were still prompted via push notification to return to the app every day to track their weight, reminded of the actions in their ‘*toolbox*’, and to plan how they could perform these actions. At the end of each week participants received a progress report and were asked to reflect. Participants could switch back to experimentation at any time. An online additional file (Additional file 3) illustrating user experience during the first week of app use, from sign up to completion of the first weekly reflection, is available at: http://figshare.com/articles/dataset/ARTEMIS_mobile_application_user_expereince_Sign_up_to_Day_9_/30104572?file=57865789. Additional file 1: Table S2 outlines intervention components mapped onto the theoretical constructs of self-regulation theory (Fig. 1B).

Participants randomised to the control received no intervention but were advised that they might wish to lose weight on their own. Contamination was mitigated by participants requiring a one-time passcode to access the ARTEMIS app, and active monitoring by the research team of multiple trial sign-ups that used the same or similar contact details or socio-demographic information.

Follow-up data were collected at 12- and 26-week intervals, while app data were collected continuously throughout the trial and collated at follow-ups. At each follow-up, participants were asked to report their weight themselves and submit a photograph of themselves, standing on the scales, showing their weight, as well as completing the same eating disorder symptoms and general health measures as at baseline, and a study-specific weight management questionnaire regarding the specific actions they used to manage their weight since the previous assessment. At the final assessment, participants were asked to rate the usefulness of the app to assist in weight loss on a scale from 1 (not useful) to 10 (very useful). Participants received payment for completing the assessments: either a £5 or £8 voucher after completing the 12-week assessment, and an £8, £12, or £20 voucher after completing the 26-week assessment (variation represents ethically approved increases in reimbursement in an attempt to improve participant retention).

### Outcomes

#### Primary outcomes

The co-primary outcomes were change in body weight from baseline to 26 weeks, and the proportion of participants achieving ≥ 5% weight loss at 26 weeks.

#### Secondary outcomes

Change in body weight from baseline to 12 weeks, and the proportion of participants achieving ≥ 5% weight loss at 12 weeks.

#### Adverse effects on symptoms of disordered eating

The proportion of participants scoring above threshold (> 7) on the abridged Eating Disorders Examination—Questionnaire Short form (EDE-QS) between baseline and 12, and 26 weeks (Additional file 4).

#### Process measures

##### Quantitative process measures

Engagement with the app was assessed through the number of days with any app engagement; daily weight readings; daily actions selected; daily action plans; weekly reflections completed; and the percentage of actions successfully completed. Both intervention and control participants reported their actions to lose weight, including the use of weight management programmes.

##### Qualitative process measures

Perceived barriers to completion of planned daily actions were assessed using free-text responses to the following question in the app: ‘Please tell us why you were unable to perform your action yesterday’. This question was asked when respondents indicated they were unable to complete their previously selected action.

### Statistical analysis

We originally aimed to recruit 1294 participants, which allowed for a 25% dropout. This gave 90% power to detect a 1.5 kg difference in mean weight change, which has previously been shown to be cost-effective [15], with a type 1 error rate of 2.5% with Bonferroni correction for co-primary outcomes. All statistical analyses were conducted in R (version 4.3.3). A statistical analysis plan was published on ClinicalTrials.gov (NCT05787652) on 2 June 2023, preceding analyses. Before analyses of outcomes, we assessed the association between baseline variables and loss to follow-up at 26 weeks. Baseline BMI and indices of multiple deprivation (IMD) were associated with loss to follow-up and included as covariates in subsequent analyses.

#### Primary analysis

We conducted intention-to-treat analyses using linear mixed effects models for repeated measures to assess weight change from baseline to 26 weeks, without imputing missing outcome data. These models account for all available weight measurements across time points under the assumption of missing at random. Fixed effects included week, treatment group, and their interaction; participant ID was included as a random effect. Thus, the primary analysis was based on a repeated measures framework rather than single time-point imputation. We used analogous logistic models to assess the proportion of participants achieving ≥ 5% loss in body weight. Pre-specified exploratory subgroup analyses were conducted by age, sex, level of educational attainment, employment status, IMD, and ethnicity.

Sensitivity analyses examined assumptions regarding missing data by (1) imputing the last-measured weight (last observation carried forward (LOCF)); (2) carrying forward the baseline weight (baseline observation carried forward (BOCF)); and (3) restricting analysis to participants with complete weight data at all time points (completer analysis). A linear mixed effects model was also used for the per-protocol analysis in which we included only participants that successfully completed at least one weigh-in and action on at least four separate weeks and had at least one action in their action toolbox. This reflected participants who had sufficiently engaged with the app to be eligible to transition as intended from the exploration to maintenance phase. A post-hoc sensitivity analysis was conducted excluding participants who did not have a valid baseline or follow-up weight verification photograph.

#### Secondary analysis

The 12-week outcomes were assessed with the primary outcome in the linear mixed effects model.

#### Adverse effects on symptoms of disordered eating

Analysis of change in the proportion of participants scoring above the threshold (> 7) on the abridged EDE-QS was assessed using mixed effects logistic regression.

#### Exploratory analysis

We assessed the actions participants used to manage their weight, including accessing other weight management programmes. These actions were classified as no action, self-help measures (alterations to diet and physical activity), and other effective measures (attending a behavioural, or using another online, weight loss programme; taking weight loss medication; or following a meal-replacement weight loss programme). A sensitivity analysis was conducted which excluded participants who used another effective strategy for weight loss. Responses to the health, quality of life, and body satisfaction questions were summarised descriptively and an ordinal logistic regression was used to compare treatment groups.

To assess whether engagement predicted weight change from baseline to 26 weeks, we used linear regression with weight change as the dependent variable and each engagement measure as a predictor in independent models. The free-text responses to the daily action completion question were analysed qualitatively using conventional content analysis. Responses were coded and then grouped into broader categories of shared meaning.

### Deviations from initial protocol

Retention was monitored and due to lower than anticipated completion rates after 20% of participants had received an initial follow-up reminder we made the following alterations following Research Ethics Committee approvals: (1) the incentive payment for 12-week follow-up was increased from £5 to £8, and for 26-week follow-up from £8 to £12, and later £20; (2) a fourth follow-up email and SMS reminder were sent to participants at 12-week follow-up to notify them of the above change; (3) we removed the requirement for photo verification at 12- and 26-week follow-up; (4) we recruited a larger sample size to account for additional drop-outs. The increases in compensation aimed to balance the trial budget with worthwhile incentives. Further, during the completion of the 26-week follow-up and prior to conducting any analyses, the final follow-up was moved following Research Ethics Committee approval from 52 to 26 weeks due to perceived low retention and the limited feasibility of increasing retention without further increasing financial incentives. No participants therefore completed any further assessments beyond the 26-week follow-up and consequently, most participants did not rate the usefulness of the app, so we were unable to analyse these data. Additionally, the definition of per-protocol analysis was altered from participants who successfully switched to maintenance as the app did not collect the necessary data to allow this. The clinicaltrials.gov registration was updated to reflect these deviations from the initial protocol.

### Role of the funding source

The funder of the study had no role in study design, data collection, data analysis, data interpretation, or writing of the report.

## Results

**Fig. 2:**
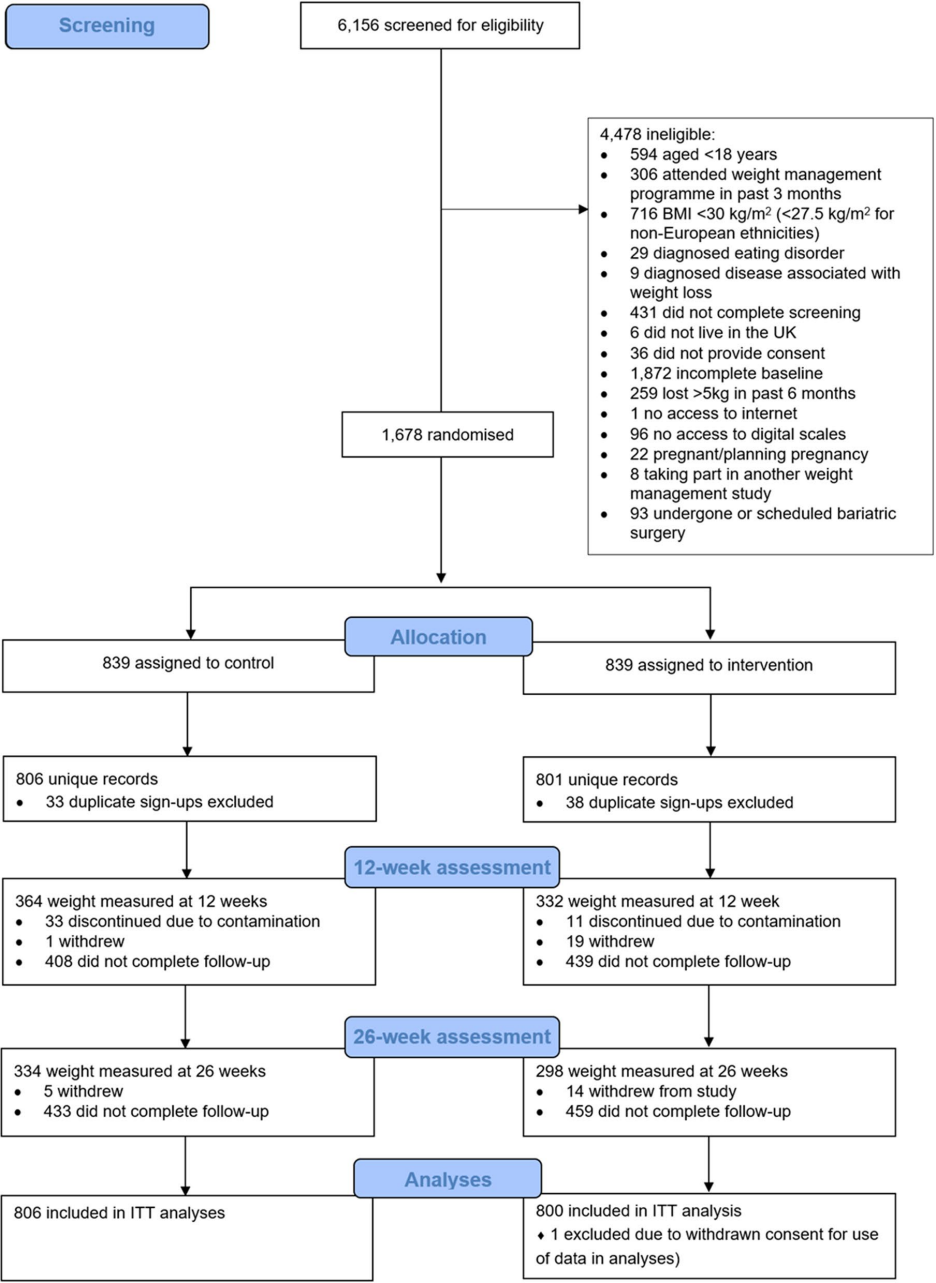
CONSORT flow diagram. Note: completion of the 26-week assessment was not dependent on the completion of the 12-week assessment

Between 13 April and 15 May 2023, 6156 screenings were conducted. Of these, 1678 records were eligible and randomly allocated to either control (*n* = 839) or intervention (*n* = 839). Duplicate sign-ups were identified and removed (*n* = 71), resulting in a final sample of 1,607 unique participants: *n* = 806 randomised to control, and *n* = 801 to intervention. Six hundred and ninety-six (43.3%) participants were followed up at 12 weeks; 632 (39.3%) at 26 weeks (Fig. 2). Overall, 48% of participants provided at least one follow-up weight reading. At 12 weeks, 489 (70%) participants followed up had a valid weight verification photograph; 528 (84%) at 26 weeks. Baseline characteristics of completers versus non-completers are available in Additional file 1: Table S3.

Mean BMI was 38 (SD: 6) kg/m^2^, and 38% scored above the threshold on the abridged disordered eating questionnaire. Mean age was 47 (SD: 11) years; most participants were female (93%), and of a white ethnic group (88%). Sixty-one per cent had an undergraduate or postgraduate degree. Further baseline characteristics are outlined in Table 1.

**Table 1:** Baseline characteristics of participants (*n* = 1606)

Characteristics	Total (*n* = 1606)	Control (*n* = 806)	Intervention (*n* = 800)*
Age, years, mean (SD)	47 (11)	46 (11)	47 (11)
Gender, *n* (%)			
Female	1499 (93.3)	743 (92.2)	756 (94.5)
Male	107 (6.7)	63 (7.8)	44 (5.5)
BMI, kg/m^2^, mean (SD)	38 (6)	38 (6)	38 (6)
Ethnicity, *n* (%)			
Asian or Asian British	85 (5.3)	46 (5.7)	39 (4.9)
Black or Black British	52 (3.2)	31 (3.8)	21 (2.6)
Mixed or multiple ethnic groups	37 (2.3)	14 (1.7)	23 (2.9)
White	1411 (88)	706 (88)	705 (88)
Other	13 (< 1)	7 (< 1)	6 (< 1)
Prefer not to say	8 (< 1)	2 (< 1)	6 (< 1)
IMD decile, *n* (%)			
1–3 (most deprived)	377 (24)	188 (24)	189 (24)
4–7	656 (42)	333 (43)	323(42)
8–10 (most affluent)	520 (33)	259 (33)	261 (34)
Missing	53 (< 1)	26 (< 1)	27 (< 1)
Highest educational qualification, *n* (%)			
No formal qualifications	27 (1.7)	13 (1.6)	14 (1.8)
GCSE/O-level	196 (12)	99 (12)	97 (12)
A levels/BTEC	373 (23)	182 (23)	191 (24)
Undergraduate/postgraduate degree	981 (61)	498 (62)	483 (60)
Prefer not to say	29 (1.8)	14 (1.7)	15 (1.9)
Employment status, *n* (%)			
Employed	1127 (70)	588 (73)	539 (67)
Self-employed	97 (6.0)	44 (5.5)	53 (6.6)
Unemployed	29 (1.8)	13 (1.6)	16 (2.0)
Looking after home and family	75 (4.7)	35 (4.3)	40 (5.0)
In education or training	34 (2.1)	16 (2.0)	18 (2.3)
Retired	148 (9.2)	62 (7.7)	86 (11)
Long-term sick or disabled	63 (3.9)	34 (4.2)	29 (3.6)
Other	33 (2.1)	14 (1.7)	19 (2.4)
Proportion scoring > 7 on EDE-QS, *n* (%)	604 (38)	286 (35)	318 (40)

*Abbreviations*: *A-level* Advanced level, *BMI* body mass index, *BTEC* business and technology education council, *EDE-QS* eating disorders examination – questionnaire short form, *GCSE* general certificate of secondary education, *IMD* index of multiple deprivation, *O-level* ordinary level, *SD* standard deviation

*One participant in the intervention group withdrew consent for use of their data

### Primary outcomes

**Fig. 3:**
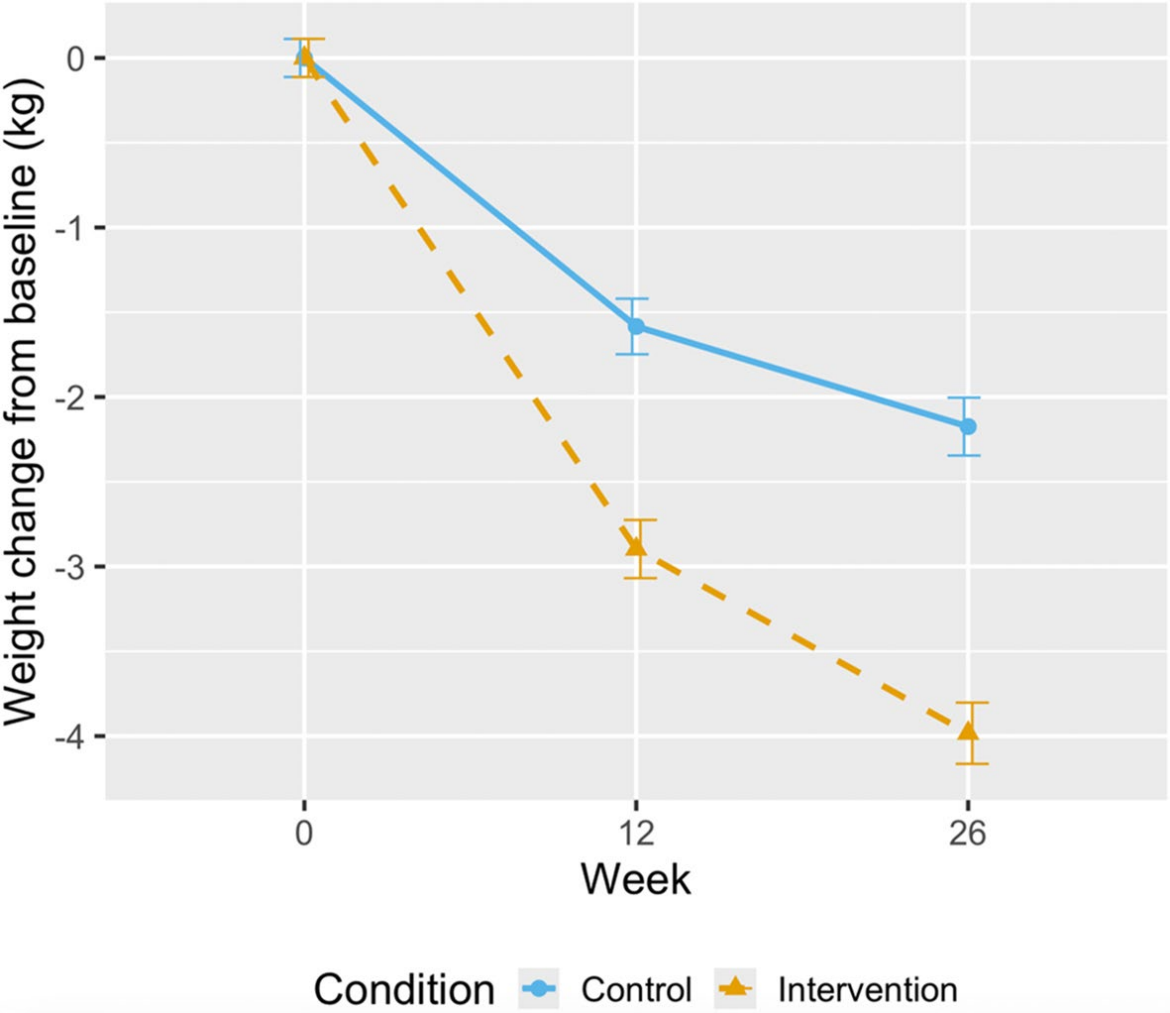
Mean weight change over 26 weeks in intention-to-treat population. Values represent mean (standard error)

Mean weight change at 26 weeks was − 3.99 (SD: 6.5) kg in the intervention, and − 2.16 (SD: 4.5) kg in the control (Fig. 3). Adjusted mean difference in weight change between the intervention and control was − 1.85 kg (95% CI: − 2.53, − 1.17; *p* < 0.001) in the intention-to-treat population using all available data in a mixed-effects model for repeated measures. Among participants followed up at 26 weeks, 34.9% of the intervention (*n* = 104) and 20.4% of the control (*n* = 68) lost ≥ 5% of their baseline body weight (adjusted odds ratio (OR): 2.11; 95% CI: 1.48, 3.03; *p* < 0.001) (Table 2). In the intervention group, 366 (46%) participants met our definition of per-protocol and analyses showed a − 2.18 kg difference between the intervention and control (95% CI: − 2.89, − 1.48; *p* < 0.001) at 26 weeks. There were 221 participants who met the definition of per-protocol and were followed up at 26 weeks; of these, 38.5% (*n* = 85) lost ≥ 5% of their baseline body weight (adjusted OR: 2.44, 95% CI: 1.67, 3.59; *p* < 0.001). There was no evidence that the effects of the intervention differed by sex, age, level of education, employment status, IMD, or ethnicity (Fig. S1 and S2). Further characteristics of per-protocol adherents versus non-adherents are available in Additional file 1: Table S4.

**Table 2:** Primary outcomes by randomised group

Timepoint	Variable	Change from baseline				Adjusted difference (95% CI)	*p* value
		Control	*n*	Intervention	*n*		
12 weeks	Mean (SD) weight change (kg)	− 1.59 (3.7)	364	− 2.88 (4.5)	332	− 1.42 (− 2.08 to − 0.77)	< 0.001
	n (%) losing ≥ 5% weight	50 (13.7)	364	88 (26.5)	332	2.27* (1.55 to 3.35)	< 0.001
26 weeks	Mean (SD) weight change (kg)	− 2.16 (4.5)	334	− 3.99 (6.5)	298	− 1.85 (− 2.53 to − 1.17)	< 0.001
	n (%) losing ≥ 5% weight	68 (20.4)	334	104 (34.9)	298	2.11* (1.48 to 3.03)	< 0.001

*Odds ratio

Sensitivity analyses on loss to follow-up using LOCF, BOCF, and completers only did not change the conclusion that the intervention led to significantly greater weight loss than control (Additional file 1: Table S5 and S6). Post hoc sensitivity analyses excluding participants who did not have a valid baseline or follow-up weight verification photograph, showed an adjusted mean difference weight change between intervention and control of − 1.95 kg (95% CI: − 2.69, − 1.20; *p* < 0.001) and 20.0% and 36.5% in the control and intervention respectively lost ≥ 5% of their baseline body weight (adjusted OR: 2.32; 95% CI: 1.57, 3.44; *p* < 0.001) (Additional file 1: Table S7).

### Secondary outcomes

At 12 weeks, the adjusted mean difference in weight change between the intervention and control groups was − 1.42 kg (95% CI: − 2.08 to − 0.77; *p* < 0.001). Among participants followed up at 12 weeks, more than twice as many participants in the intervention group lost ≥ 5% of their baseline body weight compared to the control group (adjusted OR: 2.27; 95% CI: 1.55, 3.35; *p* < 0.001).

### Adverse outcomes

The intervention group showed significantly lower odds of an EDE-QS score above the threshold (> 7) compared to the control group at both 12 and 26 weeks (Table 3). Within-person changes in abridged EDE-QS scores are reported in Additional file 1: Table S8.

**Table 3:** Adverse outcomes by randomised group

Timepoint	Variable					Adjusted difference (95% CI)	*p* value
		Control	*n*	Intervention	*n*		
12 weeks	*n* (%) > 7 on the EDE-QS	111 (29.8)	373	63 (18.6)	338	0.28* (0.16 to 0.48)	< 0.001
26 weeks	*n* (%) > 7 on the EDE-QS	84 (24.9)	337	65 (21.7)	300	0.51* (0.29 to 0.91)	0.024

*Odds ratio

### Process outcomes

#### App engagement

In the intervention group 735/801 (91.8%) participants logged into the app at least once. Overall engagement with the intervention (as measured by the total number of days with any activity), as well as greater engagement with each of the intervention components, was associated with greater weight change at 26 weeks (Table 4).

**Table 4:** ARTEMIS app engagement and association with weight change from baseline to 26 weeks per 1 standard deviation increase in each engagement measure

Engagement measure	Mean (SD)	Median (IQR)	Association with weight change at 26 weeks (*n* = 295)		
			*B*	95% CI	p*
Days with any activity	45.5 (61.7)	16 (56)	− 1.09	− 1.69, − 0.50	< 0.001
Daily weight readings	40.4 (58.2)	13 (47)	− 1.11	− 1.69, − 0.53	< 0.001
Daily actions selected	28.0 (39.9)	11 (36)	− 1.10	− 1.67, − 0.53	< 0.001
Daily action plans	27.5 (39.7)	11 (35)	− 1.11	− 1.68, − 0.53	< 0.001
Percentage of actions successfully completed	33.2 (29.5)	31.4 (59.3)	− 1.20	− 2.00, − 0.41	0.003
Weekly reflections	7.7 (10.6)	2 (11)	− 1.02	− 1.64, − 0.40	0.001

*Significance defined based on a Bonferroni-corrected *p* value of 0.0083 (0.05/6)

#### Actions used to manage weight

At 26 weeks, 337 (41.8%) control participants and 300 (37.5%) intervention participants reported on the actions they used to lose weight since their previous assessment (Additional file 1: Table S9). Eighty (26.7%) intervention participants reported using the ARTEMIS app, and 87 (29%) reported using strategies learned from the ARTEMIS app but were not actively using the app. Overall, between the 12- and 26-week follow-up, almost twice as many participants in the intervention (61.3%) compared to control (32.9%) took effective action to lose weight (including in the intervention using the ARTEMIS app or learned strategies from the app). Pre-specified sensitivity analysis excluding participants who used other effective strategies for weight loss showed an adjusted mean difference in weight change between intervention and control of −2.40 kg (95% CI: −3.27, − 1.53 kg; *p* < 0.001) at 26 weeks. Similarly, 37.2% of the intervention group and 14.4% of the control group lost ≥ 5% of their baseline body weight (adjusted OR: 3.53; 95% CI: 2.14, 5.97; *p* < 0.001) (Additional file 1: Table S10).

#### Self-reported barriers to planned daily action completion

Within the intervention group, there were 2399 valid responses provided to the question regarding reasons for daily action incompletion. The most common barriers to performing daily weight loss actions were as follows: (1) being too busy/not having enough time; 2) forgetting; 3) work commitments; and 4) eating out or social plans (Additional file 1: Table S11).

#### Self-reported rating on health, quality of life, and body satisfaction

At 26 weeks, there was no evidence of a difference in self-reported rating of health (OR: 1.27; 95% CI: 0.96, 1.70; *p* = 0.09) or quality of life (OR: 1.13; 95% CI: 0.85, 1.50; *p* = 0.41) between the intervention and control. The odds of reporting higher body satisfaction were significantly higher in the intervention group compared to the control at 12 (OR: 1.88; 95% CI: 1.42, 2.50; *p* < 0.001), and 26 weeks (OR: 1.80; 95% CI: 1.35, 2.42; *p* < 0.001) (Additional file 1: Table S12).

## Discussion

Our self-regulation theory-based mobile application led to 1.85 kg greater weight loss, doubled the odds of losing ≥ 5% body weight, halved the odds of symptoms of disordered eating, and improved body image compared with no support to lose weight at six months. Importantly, the intervention was entirely automated with no in-person coaching or support. The intervention appears to achieve its effects through engagement with the app, which participants partially used in favour of other effective weight loss strategies that were more commonly used by control participants.

Previous trials of weight loss interventions with a digital component and more intensive in-person or remote support have proven to be effective in supporting weight loss among adults living with excess weight or obesity, and hypertension or diabetes, with a mean weight loss of − 2.9 kg (95% CI: − 3.5, − 2.3) in intervention compared to 1.0 kg (95% CI: − 1.9, − 0.1) in usual care control at six months [16]. Similarly, a web-based weight loss intervention with remote nurse support showed an additional − 1.97 kg weight loss compared to normal care control among adults living with obesity and hypertension, high cholesterol, or diabetes [17]. However, there has been scepticism surrounding the effectiveness of low or very low intensity interventions [18, 19]. Our entirely self-managed mobile application which required no professional input or other healthcare resources, produced broadly comparable weight loss to these more resource-intensive interventions.

Participants in the control group who were followed up lost over 2 kg, somewhat larger than is typical for minimal intervention control groups in weight loss trials [20]. Moreover, a third reported using effective weight loss interventions, approximately three times greater than a population of people living with obesity unselected by motivation to lose weight and where follow-up rates were much higher [21]. This suggests control group participants may have been more motivated than usual to lose weight, which is likely reflective of the nature of our recruitment methods. For those who were seeking support for weight loss, the intervention led to greater weight loss, and less recourse to other weight loss activities, than in people left to lose weight without support. Those in the control group were more likely to either self-fund or seek other publicly funded known effective weight loss interventions, underscoring the intervention’s efficacy in promoting self-directed weight management efforts. The effectiveness of the app among populations that may be less motivated to lose weight, or engage in self-referral, is of interest for future research (e.g. in the context of in-person offers of the app within brief opportunistic interventions in healthcare settings).

When offered to a general population sample there was no evidence that there was an adverse effect on symptoms of disordered eating, despite fears that a focus on self-regulation might encourage obsessive or over-controlling thoughts and actions. There was evidence of a reduction in symptoms of disordered eating and improved body satisfaction at three and six months, in a sample with higher-than-average symptoms of disordered eating at baseline (which may be explained by our recruitment method) [12]. This contributes to growing evidence indicating behavioural weight management interventions may reduce eating disorder symptoms [22, 23]. However, we used an abridged (six-item) version of the EDE-QS, removing items that would be likely to increase when people living with obesity engage in a weight loss attempt. We did so to increase the specificity of the questionnaire to true changes in concerning symptoms of eating disorders within the context of this weight loss trial. A limitation of our approach is that to align with the reduction in items from 12 to six, we applied an ad hoc cutoff of > 7 as a proportional reduction from the established EDE-QS threshold of 15, representing a logical rather than a previously validated cutoff. Our findings are consistent with a recent systematic review and meta-analysis examining the impact of weight loss interventions on disordered eating symptoms in people living with excess weight and obesity, which found that weight loss interventions consistently improved disordered eating scores [24]. Our abridged EDE-QS could be used in other weight management trials to indicate the occurrence of disordered eating.

We found that engagement with the intervention components was associated with greater weight loss. Previous research has shown that few people naturally complete the self-regulation process despite being prompted by self-monitoring, and that action planning was associated with weight loss but was implemented rarely [10]. Further, participants who reflected on their behaviour were more likely to complete the other stages of the self-regulation process. Our results suggest that guiding individuals through the self-regulation process, and greater engagement with each component of the self-regulatory cycle, improves weight loss. Additionally, we also add definitive evidence that interventions using self-regulation can be effective for weight loss [11, 25, 26]. Engagement with the app was modest, with 46% of participants meeting per-protocol criteria, and engagement declining over time. While this is not unusual for digital interventions, outside of a trial, the ARTEMIS app is most likely to attract people who are motivated to lose weight and digitally enabled. The aim of the trial was to investigate the effectiveness of an entirely self-managed scalable intervention. This type of intervention requires digital literacy, motivation to use a mobile application, and a high level of individual agency. As such it is not likely to be suitable for everyone; however, many people are trying to self-manage their weight, are increasingly digitally literate, and could benefit from an evidence-based digital intervention. Our PPI group suggested future improvements on the app, including in-app step tracking, integration with wearable devices, and access to personalised coaching. Exploring effective strategies to improve and sustain engagement, such as in-app step tracking, integration with wearable devices, tailored reminders, gamification elements, social or peer support features, and access to personalised coaching is of potential interest for future research.

The ARTEMIS app provides an effective, safe, low-cost, and scalable weight loss treatment option for adults living with obesity who wish to lose weight. We found no evidence that the treatment effect on weight loss at six months differed by age, sex, educational attainment, employment status, IMD, or ethnicity, suggesting it would not worsen existing health inequalities and by providing free access to an effective weight-loss intervention it could reduce disparities in access to treatment. Further, most participants were female and while we included a somewhat representative sample of minority ethnic groups, they form a small minority of the middle-aged and older population, meaning these exploratory subgroup analyses were underpowered to detect such differences. Our findings may not be broadly generalisable to males and specific ethnic groups but do reflect the population seeking app-based support for weight loss. This is consistent with weight management trials generally [27, 28] and digital weight management trials specifically [29].

A major strength of this trial was the rapid large-scale recruitment of participants. We did however anticipate that retention may be a challenge and ensured that demands of follow-up were low and compensated individuals for their time. Despite this, 40% were followed up at six months and 48% provided at least one weight reading after baseline. The mixed models for repeated measures allowed use of all available data with missing data implicitly included as missing at random. Sensitivity analyses examining assumptions regarding missing data did not change the conclusions that the intervention was effective but did reduce the size of the intervention effect. Our follow-up rates were low compared to clinical trials with selected participants and in-person contact, but typical of trials of digital interventions, where attrition rates range from 9 to 86% [30] and from 9 to 89% in digital weight loss interventions specifically [31]. Nevertheless, the rate of attrition is a critical limitation which may overestimate the effectiveness of the intervention if participants who were not as successful did not complete follow-up. The similar rate of passive-driven attrition (i.e. loss to follow-up) between intervention and control, occurring predominantly between baseline and 12-week follow-up, suggests that our recruitment method, while increasing the external validity of our findings, predominantly contributed to the rate of attrition through being open to all eligible persons regardless of their commitment to the trial, and with only moderate barriers to progressing to randomisation. This is partially mitigated by our BOCF/LOCF analyses. However, given its reach and lack of resource requirements, even if a modest proportion of those who considered the mobile application continue to use it, this could have a large population impact. The most pertinent future direction for the ARTEMIS app is to be made publicly available, with implementation research monitoring long-term usage and weight change outside the context of the trial.

Despite the large number of mobile applications supporting weight loss, many lack theoretical grounding or substantial evidence of effectiveness beyond anecdotal reports or observational studies. Here, we conducted a large, pragmatic, randomised controlled trial, which provides robust real-world evidence on the expected benefits of the intervention. The intervention was found to be effective without adverse effects, meaning it could be offered through healthcare providers or elsewhere publicly or privately to people who are seeking self-managed support for weight loss.

## Conclusions

An app which promoted self-regulation with no in-person support, was successfully implemented in a real-world setting and increased weight loss and reduced symptoms of disordered eating among adults living with obesity who were seeking weight loss support.

## Supplementary Information

Additional file 1: Supplementary Tables S1-S12/Supplementary Figures S1-S2.

Additional file 2: Overview of the weight loss action categories, individual weight loss actions, “What to do” and “Why does it matter?” descriptions, and associated tips, in the ARTEMIS mobile application.

Additional file 3: ARTEMIS mobile application user experience video (from sign up to Day 9). This additional file illustrates user experience during the first week of ARTEMIS mobile application use, from sign up to completion of the first weekly reflection https://figshare.com/articles/dataset/ARTEMIS_mobile_application_user_expereince_Sign_up_to_Day_9_/30104572?file=57865789.

Additional file 4: Electronic case report form for the ARTEMIS trial, including screening, baseline, and follow-up assessments.

## Data Availability

All study data will be made available upon reasonable request.

## Abbreviations

ARC: Applied Research Collaboration
ARTEMIS: Adults Regulating Their weight Everyday with Mobile Internet Support
BOCF: Baseline observation carried forward
BMI: Body mass index
BRC: Biomedical Research Centre
BTEC: Business and Technology Education Council
CI: Confidence interval
EDE-QS: Eating Disorders Examination – Questionnaire Short form
GCSE: General Certificate of Secondary Education
IMD: Index of multiple deprivation
iCASE: Industrial Collaborative Award in Science and Engineering
LOCF: Last observation carried forward
MRC: Medical Research Council
NHS: National Health Service
NIHR: National Institute for Health and Care Research
OR: Odds ratio
PIS: Participant information sheet
PPI: Patient and Public Involvement
REDCap: Research Electronic Database Capture
SD: Standard deviation

## References

[1] Vos T, Allen C, Arora M, Barber RM, Bhutta ZA, Brown A, et al. Global, regional, and national incidence, prevalence, and years lived with disability for 310 diseases and injuries, 1990–2015: a systematic analysis for the Global Burden of Disease Study 2015. Lancet. 2016;388(10053):1545–602. 27733282 10.1016/S0140-6736(16)31678-6 PMC5055577

[2] Welsh CE, Matthews FE, Jagger C. Trends in life expectancy and healthy life years at birth and age 65 in the UK, 2008–2016, and other countries of the EU28: an observational cross-sectional study. Lancet Reg Health–Eur. 2021;2:100023. 10.1016/j.lanepe.2020.100023 PMC8042672 33870247

[3] Lobstein T, Jackson-Leach R, Powis J, Brinsden H, Gray M. World obesity atlas 2023. 2023.

[4] Di Angelantonio E, Bhupathiraju SN, Wormser D, Gao P, Kaptoge S, De Gonzalez AB, et al. Body-mass index and all-cause mortality: individual-participant-data meta-analysis of 239 prospective studies in four continents. Lancet. 2016;388(10046):776–86. 27423262 10.1016/S0140-6736(16)30175-1 PMC4995441

[5] Murray CJL, Aravkin AY, Zheng P, Abbafati C, Abbas KM, Abbasi-Kangevari M, et al. Global burden of 87 risk factors in 204 countries and territories, 1990–2019: a systematic analysis for the Global Burden of Disease Study 2019. Lancet. 2020;396(10258):1223–49. 33069327 10.1016/S0140-6736(20)30752-2 PMC7566194

[6] Office for National Statistics, (UK). Internet users, UK: 2020 - Internet use in the UK; annual estimates by age, sex, disability and geographical location. 2020 URL: https://www.ons.gov.uk/businessindustryandtrade/itandinternetindustry/bulletins/internetusers/2020. Accessed: 08/04/2024.

[7] Islam MM, Poly TN, Walther BA, Li Y-C. Use of mobile phone app interventions to promote weight loss: meta-analysis. JMIR mHealth uHealth. 2020;8(7):e17039. 32706724 10.2196/17039 PMC7407260

[8] Madigan CD, Jolly K, Lewis AL, Aveyard P, Daley AJ. A randomised controlled trial of the effectiveness of self-weighing as a weight loss intervention. Int J Behav Nutr Phys Act. 2014;11(1):125. 10.1186/s12966-014-0125-9 PMC4195875 25301251

[9] Madigan CD, Daley AJ, Lewis AL, Aveyard P, Jolly K. Is self-weighing an effective tool for weight loss: a systematic literature review and meta-analysis. Int J Behav Nutr Phys Act. 2015;12:1–11. 26293454 10.1186/s12966-015-0267-4 PMC4546162

[10] Frie K, Hartmann-Boyce J, Pilbeam C, Jebb S, Aveyard P. Analysing self-regulatory behaviours in response to daily weighing: a think-aloud study with follow-up interviews. Psychol Health. 2020;35(1):16–35. 31198059 10.1080/08870446.2019.1626394 PMC6961301

[11] Frie K, Hartmann-Boyce J, Jebb SA, Aveyard P. Effectiveness of a self-regulation intervention for weight loss: a randomized controlled trial. Br J Health Psychol. 2020;25(3):652–76. 10.1111/bjhp.12436 32489005

[12] Prnjak K, Mitchison D, Griffiths S, Mond J, Gideon N, Serpell L, et al. Further development of the 12-item EDE-QS: identifying a cut-off for screening purposes. BMC Psychiatry. 2020;20(1):146. 10.1186/s12888-020-02565-5 PMC7118929 32245441

[13] Gideon N, Hawkes N, Mond J, Saunders R, Tchanturia K, Serpell L. Development and psychometric validation of the EDE-QS, a 12 item short form of the eating disorder examination questionnaire (EDE-Q). PLoS ONE. 2016;11(5):e0152744. 10.1371/journal.pone.0152744 PMC4854480 27138364

[14] Frie K, Hartmann-Boyce J, Jebb SA, Aveyard P. Testing the effectiveness of a weight loss intervention to enhance self-regulation in adults who are obese: protocol for a randomised controlled trial. BMJ Open. 2019;9(12):e031572. 31818839 10.1136/bmjopen-2019-031572 PMC6924834

[15] Retat L, Pimpin L, Webber L, Jaccard A, Lewis A, Tearne S, et al. Screening and brief intervention for obesity in primary care: cost-effectiveness analysis in the BWeL trial. Int J Obes (Lond). 2019;43(10):2066–75. 10.1038/s41366-018-0295-7 PMC6776469 30705390

[16] Baer HJ, Rozenblum R, De La Cruz BA, Orav EJ, Wien M, Nolido NV, et al. Effect of an online weight management program integrated with population health management on weight change: a randomized clinical trial. JAMA. 2020;324(17):1737–46. 33141209 10.1001/jama.2020.18977 PMC7610192

[17] Little P, Stuart B, Hobbs FR, Kelly J, Smith ER, Bradbury KJ, et al. An internet-based intervention with brief nurse support to manage obesity in primary care (POWeR+): a pragmatic, parallel-group, randomised controlled trial. Lancet Diabetes Endocrinol. 2016;4(10):821–8. 27474214 10.1016/S2213-8587(16)30099-7

[18] Neve M, Morgan PJ, Jones PR, Collins CE. Effectiveness of web-based interventions in achieving weight loss and weight loss maintenance in overweight and obese adults: a systematic review with meta-analysis. Obes Rev. 2010;11(4):306–21. 10.1111/j.1467-789X.2009.00646.x 19754633

[19] Wadden TA, Tronieri JS, Butryn ML. Lifestyle modification approaches for the treatment of obesity in adults. Am Psychol. 2020;75(2):235–51. 10.1037/amp0000517 PMC7027681 32052997

[20] Johns DJ, Hartmann-Boyce J, Jebb SA, Aveyard P. Weight change among people randomized to minimal intervention control groups in weight loss trials. Obesity. 2016;24(4):772–80. 27028279 10.1002/oby.21255 PMC4820081

[21] Aveyard P, Lewis A, Tearne S, Hood K, Christian-Brown A, Adab P, et al. Screening and brief intervention for obesity in primary care: a parallel, two-arm, randomised trial. Lancet. 2016;388(10059):2492–500. 27789061 10.1016/S0140-6736(16)31893-1 PMC5121130

[22] Jebeile H, Libesman S, Melville H, Low-Wah T, Dammery G, Seidler AL, et al. Eating disorder risk during behavioral weight management in adults with overweight or obesity: a systematic review with meta-analysis. Obes Rev. 2023;24(6):e13561. 10.1111/obr.13561 PMC10909435 36919475

[23] Benn Y, Webb TL, Chang BPI, Harkin B. What is the psychological impact of self-weighing? A meta-analysis. Health Psychol Rev. 2016;10(2):187–203. 26742706 10.1080/17437199.2016.1138871 PMC4917920

[24] Tsompanaki E, Koutoukidis DA, Wren G, Tong H, Theodoulou A, Wang D, et al. The impact of weight loss interventions on disordered eating symptoms in people with overweight and obesity: a systematic review & meta-analysis. EClinicalMedicine. 2025;80:103049. 10.1016/j.eclinm.2024.103049 PMC11841075 39981343

[25] Michie S, Abraham C, Whittington C, McAteer J, Gupta S. Effective techniques in healthy eating and physical activity interventions: a meta-regression. Health Psychol. 2009;28(6):690–701. 19916637 10.1037/a0016136

[26] Kliemann, Croker, Johnson, Beeken. Development of the top tips habit-based weight loss app and preliminary indications of its usage, effectiveness, and acceptability: mixed-methods pilot study. JMIR Mhealth Uhealth. 2019;7(5):e12326. 31094352 10.2196/12326 PMC6533874

[27] Haughton CF, Silfee VJ, Wang ML, Lopez-Cepero AC, Estabrook DP, Frisard C, et al. Racial/ethnic representation in lifestyle weight loss intervention studies in the United States: a systematic review. Prev Med Rep. 2018;9:131–7. 10.1016/j.pmedr.2018.01.012 PMC5880332 29616185

[28] Pagoto SL, Schneider KL, Oleski JL, Luciani JM, Bodenlos JS, Whited MC. Male inclusion in randomized controlled trials of lifestyle weight loss interventions. Obesity. 2012;20(6):1234–9. 21633403 10.1038/oby.2011.140

[29] Patel ML, Wakayama LN, Bennett GG. Self-monitoring via digital health in weight loss interventions: a systematic review among adults with overweight or obesity. Obesity. 2021;29(3):478–99. 33624440 10.1002/oby.23088 PMC12838191

[30] Meyerowitz-Katz G, Ravi S, Arnolda L, Feng X, Maberly G, Astell-Burt T. Rates of attrition and dropout in app-based interventions for chronic disease: systematic review and meta-analysis. J Med Internet Res. 2020;22(9):e20283. 10.2196/20283 PMC7556375 32990635

[31] Hutchesson MJ, Rollo ME, Krukowski R, Ells L, Harvey J, Morgan PJ, et al. eH ealth interventions for the prevention and treatment of overweight and obesity in adults: a systematic review with meta-analysis. Obes Rev. 2015;16(5):376–92. 25753009 10.1111/obr.12268

