# Supplementary material for: The effectiveness and safety of a mobile application-based self-regulation intervention to support weight loss among adults living with obesity: a large-scale pragmatic randomised controlled trial
Source: BMC Med. 2025 Nov 29;24:6. doi: 10.1186/s12916-025-04519-8 (PMC12771856; doi:10.1186/s12916-025-04519-8)
Supplement: Supplementary file 1 — Additional file 1: Supplementary Tables S1-S12/Supplementary Figures S1-S2. [file 12916_2025_4519_MOESM1_ESM.docx]

**Additional file 1.**

**Supplementary tables S1-S12 / Supplementary Figures S1-S2**

*Table S1: Full eligibility criteria*

| **Inclusion** | **Exclusion** |
| --- | --- |
| - Aged ≥18 years; - Body mass index ≥30 kg/m2 (≥27·5 kg/m2 for non-European ethnicities); - Full-time resident in the UK and not intending to move outside the UK in the next 12 months; - Able to access the internet with a smartphone or tablet; - Able to access and use a digital weighing scale. | - Unable to understand the English language; - Currently signed up with an intention to attend, or had in the previous three months attended, a weight management programme; - Currently participating in another weight management study; - Lost >5kg in the previous six months; - Previously undergone bariatric surgery or scheduled to have bariatric surgery; - Currently pregnant or planning pregnancy in the next 12 months; - Currently or previously diagnosed with an eating disorder; - Recently diagnosed with a disease, or expected to undergo treatment for a disease, associated with substantial weight loss e.g., cancer treatment. |

Table S2: ARTEMIS intervention components mapped onto the theoretical constructs of self-regulation theory.

| Theoretical construct | ARTEMIS intervention component |
| --- | --- |
| Goal setting | Setting (and refining) a goal body weight in the ARTEMIS app. Calculated at sign up as participant’s current weight minus the weight they would like to lose in the next two months. Once achieved participants were prompted to enter a new goal weight. A participants’ goal weight could be viewed and edited at any time in the ‘progress’ tab of the app. |
| Self-monitoring | Exploration: Daily self-weighing and recording of action plan success status. Maintenance: Daily self-weighting and weekly recording of performance of actions in toolbox. |
| Action planning | Exploration: Weekly choosing a weight loss action category and daily choosing a weight loss action from that category to perform, including details about how, when, and where the action will be performed that day. Maintenance: A daily reminder about the weight loss actions in participants’ toolbox. |
| Feedback | Weekly feedback on weight loss progress. |
| Reflection | Daily reflection on weight loss action success status, including when unsuccessful detailing why participant’s thought they were unable to perform the action, and if there is anything they think would be useful to do differently next time they choose the action. Weekly reflections on behaviour throughout the week, including prompts to participants on their happiness with their progress during the week and why they thought their weight changed the way it did. |
| Self-efficacy | Exploration: Each day participants were asked if they would select the weight loss action from the previous day again. At the end of the week, participants were presented with all of the weight loss actions they said they would choose again and asked if they wanted to add these to their action toolbox of actions that worked for them, that they would like to use to control their weight going forward; over time building a collect of actions that work for them.  Maintenance: Once participants moved to the maintenance phase, they were exclusively using the actions in their toolbox which they determined work for them. |

*Table S3: Baseline characteristics of non-completers (n=1,309) vs completers (n=567).*

| Characteristics | Total  (n=1,606) | Non-completers (n=1,039) | Completers* (n=567) | P-value |
| --- | --- | --- | --- | --- |
| Age, years, mean (SD) | 47 (11) | 47 (11) | 46 (11) | 0.3 |
| Gender, n (%) 0.4 | | | | |
| Female | 1,499 (93·3) | 974 (93·7) | 525 (92·6) |  |
| Male | 107 (6·7) | 65 (6·3) | 42 (7·4) |  |
| BMI, kg/m^2^, mean (SD) | 38 (6) | 38 (6) | 37 (6) | 0.002 |
| Ethnicity, n (%) 0.05 | | | | |
| Asian or Asian British | 85 (5·3) | 58 (5·6) | 27 (4·8) |  |
| Black or Black British | 52 (3·2) | 41 (3·9) | 11 (1·9) |  |
| Mixed or multiple ethnic groups | 37 (2·3) | 19 (1·8) | 18 (3·2) |  |
| White | 1,411 (88) | 904 (87) | 507 (89) |  |
| Other | 13 (<1) | 11 (1) | 2 (<1) |  |
| Prefer not to say | 8 (<1) | 6 (<1) | 2 (<1) |  |
| IMD decile, n (%)  0.2 | | | | |
| 1-3 (most deprived) | 377 (24) | 258 (26) | 119 (22) |  |
| 4-7 | 656 (42) | 423 (42) | 233 (43) |  |
| 8-10 (most affluent) | 520 (33) | 325 (32) | 195 (36) |  |
| Missing | 53 (<1) | 33 (<1) | 20 (<1) |  |
| Highest Educational Qualification, n (%) 0.009 | | | | |
| No formal qualifications | 27 (1·7) | 20 (1·9) | 7 (1·2) |  |
| GCSE/O-level | 196 (12) | 143 (14) | 53 (9.3) |  |
| A levels/BTEC | 373 (23) | 250 (24) | 123 (22) |  |
| Undergraduate/postgraduate degree | 981 (61) | 604 (58) | 377 (66) |  |
| Prefer not to say | 29 (1·8) | 22 (1·9) | 7 (1·2) |  |
| Employment Status, n (%) 0.1 | | | | |
| Employed | 1,127 (70) | 740 (71) | 387 (68) |  |
| Self-employed | 97 (6·0) | 64 (6·2) | 33 (5·8) |  |
| Unemployed | 29 (1·8) | 21 (2·0) | 8 (1·4) |  |
| Looking after home and family | 75 (4·7) | 43 (4·1) | 32 (5·6) |  |
| In education or training | 34 (2·1) | 27 (2·6) | 7 (1·2) |  |
| Retired | 148 (9·2) | 83 (8·0) | 65 (11) |  |
| Long-term sick or disabled | 63 (3·9) | 42 (4·0) | 21 (3·7) |  |
| Other | 33 (2·1) | 19 (1·8) | 14 (2·5) |  |
| Proportion scoring >7 on EDE-QS,  n (%) | 604 (38) | 403 (39) | 201 (35) | 0.2 |

**Completers are defined as participants who had weight data available at all time points.*

*Abbreviations: A-level: Advanced level; BMI: body mass index; BTEC: business and technology education council; EDE-QS: eating disorders examination – questionnaire short form; GCSE: general certificate of secondary education; IMD: index of multiple deprivation; O-level: ordinary level; SD: standard deviation.*

*P values were calculated using 2-tailed t tests for continuous variables or chi-squared tests for categorical variables to compare differences between completers and non-completers.*

*Table S4: Baseline characteristics of non-adherents (n=434) vs per-protocol adherents (n=n=366) intervention participants.*

| Characteristics | Intervention (n=800) | Non-adherents (n=434) | Per-protocol adherents* (n=366) | P-value |
| --- | --- | --- | --- | --- |
| Age, years, mean (SD) | 47 (11) | 46 (12) | 48 (11) | 0.009 |
| Gender, n (%) 0.7 | | | | |
| Female | 756 (94.5) | 409 (94.2) | 347 (94.8) |  |
| Male | 44 (5.5) | 25 (5.8) | 19 (5.2) |  |
| BMI, kg/m^2^, mean (SD) | 38 (6.1) | 38 (6.1) | 37 (6.1) | 0.07 |
| Ethnicity, n (%) 0.03 | | | | |
| Asian or Asian British | 39 (4.9) | 25 (5.8) | 14 (3.8) |  |
| Black or Black British | 21 (2.6) | 17 (3.9) | 4 (1.1) |  |
| Mixed or multiple ethnic groups | 23 (2.9) | 15 (3.5) | 8 (2.2) |  |
| White | 705 (88) | 369 (85) | 336 (92) |  |
| Other | 6 (<1) | 5 (1.2) | 1 (<1) |  |
| Prefer not to say | 6 (<1) | 3 (<1) | 3 (<1) |  |
| IMD decile, n (%)  0.07 | | | | |
| 1-3 (most deprived) | 189 (24) | 116 (28) | 73 (21) |  |
| 4-7 | 323 (42) | 171 (41) | 152 (43) |  |
| 8-10 (most affluent) | 261 (34) | 133 (32) | 128 (36) |  |
| Missing | 27 (<1) | 14 (<1) | 13 (<1) |  |
| Highest Educational Qualification, n (%) <0.001 | | | | |
| No formal qualifications | 14 (1.8) | 13 (3.0) | 1 (0.3) |  |
| GCSE/O-level | 97 (12) | 66 (15) | 31 (8.5) |  |
| A levels/BTEC | 191 (24) | 105 (24) | 86 (23) |  |
| Undergraduate/postgraduate degree | 483 (60) | 239 (55) | 244 (67) |  |
| Prefer not to say | 15 (1.9) | 11 (2.5) | 4 (1.1) |  |
| Employment Status, n (%) 0.01 | | | | |
| Employed | 539 (67) | 306 (71) | 233 (64) |  |
| Self-employed | 53 (6.6) | 25 (5.8) | 28 (7.7) |  |
| Unemployed | 16 (2.0) | 13 (3.0) | 3 (<1) |  |
| Looking after home and family | 40 (5.0) | 20 (4.6) | 20 (5.5) |  |
| In education or training | 18 (2.3) | 10 (2.3) | 8 (2.2) |  |
| Retired | 86 (11) | 36 (8.3) | 50 (14) |  |
| Long-term sick or disabled | 29 (3.6) | 18 (4.1) | 11 (3.0) |  |
| Other | 19 (2.4) | 6 (1.4) | 13 (3.6) |  |
| Proportion scoring >7 on EDE-QS,  n (%) | 318 (40) | 185 (43) | 133 (36) | 0.07 |

**One participant in the intervention group withdrew consent for use of their data. Per-protocol adherents were defined as participants that successfully completed a minimum of one weigh-in and action on at least four separate weeks and had at least one action in their toolbox.*

*Abbreviations: A-level: Advanced level; BMI: body mass index; BTEC: business and technology education council; EDE-QS: eating disorders examination – questionnaire short form; GCSE: general certificate of secondary education; IMD: index of multiple deprivation; O-level: ordinary level; SD: standard deviation.*

*P values were calculated using 2-tailed t tests for continuous variables or chi-squared tests for categorical variables to compare differences between non-adherents and per-protocol adherents.*

*Table S5: Adjusted difference in weight between treatment groups under different missing data approaches.*

|  |  | **BOCF** | | **LOCF** | | **Completers only** | |
| --- | --- | --- | --- | --- | --- | --- | --- |
|  |  | Control (n=806) | Intervention (n=800) | Control (n=806) | Intervention (n=800) | Control (n=301) | Intervention (n=266) |
| **12 weeks** | Mean (SD) weight change | -0·72 (2·61) | -1·19 (3·22) | -0·72 (2·61) | -1·19 (3·22) | -1·53 (3·68) | -2·92 (4·55) |
|  | Adjusted difference* (95% CI) | -0·51 (-0·83 to -0·19) | | -0·48 (-0·79 to -0·16) | | -1·49 (-2·23 to -0·73) | |
|  | p-value | 0·002 | | 0·003 | | <0·001 | |
| **26 weeks** | Mean (SD) weight change | -0·9 (3·09) | -1·49 (4·39) | -1·04 (3·26) | -1·71 (4·54) | -2·19 (4·6) | -3·86 (6·44) |
|  | Adjusted difference*  (95% CI) | -0·65 (-0·97 to -0·33) | | -0·67 (-0·98 to -0·35) | | -1·78 (-2·54 to -1·03) | |
|  | p-value | <0·001 | | <0·001 | | <0·001 | |

** Adjusted mean difference (kg) (95% CI) calculated using linear mixed effects models with fixed effects for condition, week, and group*week interaction, and a random effect for participant. IMD and baseline BMI were included as covariates as baseline values were predictive of missingness in the data.*

*Table S6: Adjusted odds of losing >5% baseline body weight between treatment groups under different missing data approaches.*

|  |  | **BOCF** | | **LOCF** | | **Completers only** | |
| --- | --- | --- | --- | --- | --- | --- | --- |
|  |  | Control (n=806) | Intervention (n=800) | Control (n=806) | Intervention (n=800) | Control (n=301) | Intervention (n=266) |
| **12 weeks** | n (%) losing ≥5% weight | 50 (6·2) | 88 (11·0) | 50 (6·2) | 88 (11·0) | 40 (13·3) | 74 (27·8) |
|  | Odds ratio* (95% CI) | 1·97 (1·36 to 2·89) | | 1·86 (1·30 to 2·70) | | 2·77 (1·78 to 4·35) | |
|  | p-value | <0·001 | | <0·001 | | <0·001 | |
| **26 weeks** | n (%) losing ≥5% weight | 68 (8·4) | 104 (13·0) | 78 (9·7) | 118 (14·8) | 62 (20·6) | 90 (33·8) |
|  | Odds ratio*  (95% CI) | 1·69 (1·21 to 2·37) | | 1·61 (1·19 to 2·19) | | 2·05 (1·39 to 3·04) | |
|  | p-value | 0·002 | | 0·002 | | <0·001 | |

** Adjusted odds ratio (95% CI) calculated using logistic regression models. IMD and baseline BMI were included as covariates as baseline values were predictive of missingness in the data.*

*Table S7: Sensitivity analysis excluding those who did not have a valid baseline weight verification photo and did not have a valid follow-up weight verification photo.*

| Timepoint | Variable | Mean (SD) change from baseline | | | | Adjusted difference  (95% CI) | *P value* |
| --- | --- | --- | --- | --- | --- | --- | --- |
|  |  | Control | n | Intervention | n |  |  |
| 12 weeks | Weight (kg) | -1·54 (3·6) | 290 | -2·90 (4·3) | 262 | -1·41*  (-2·15 to -0·67) | <0·001 |
|  | n (%) losing ≥5% weight | 40 (13·8) | 290 | 72 (27·5) | 262 | 2·36†  (1·54 to 3·67) | <0·001 |
| 26 weeks | Weight (kg) | -2·14 (4·4) | 280 | -4·12 (6·5) | 249 | -1·95*  (-2·69 to -1·20) | <0·001 |
|  | N (%) lost ≥5% weight | 56 (20·0) | 280 | 91 (36·5) | 249 | 2·32†  (1·57 to 3·44) | <0·001 |

**Adjusted mean difference (kg) (95% CI) calculated using linear mixed effects models with fixed effects for condition, week and group*week interaction, and a random effect for participant. IMD and baseline BMI were included as covariates as baseline values were predictive of missingness in the data.*

*†Odds ratio.*

| **Timepoint** | **Group** | **Increased EDE-QS** | **Reduced EDE-QS** | **Unchanged EDE-QS** | **Missing EDE-QS** |
| --- | --- | --- | --- | --- | --- |
| 12 weeks | Control | 154 (19.1%) | 152 (18.9%) | 67 (8.3%) | 433 (53.7%) |
| 12 weeks | Intervention | 81 (10.1%) | 193 (24.1%) | 64 (8.0%) | 462 (57.8%) |
| 24 weeks | Control | 118 (14.6%) | 163 (20.2%) | 56 (6.9%) | 469 (58.2%) |
| 24 weeks | Intervention | 75 (9.4%) | 180 (22.5%) | 45 (5.6%) | 500 (62.5%) |

*Table S8: Within person changes in EDE-QS score.*

*Table S9: Participant self-reported actions* used to manage weight at 12- and 26-weeks.*

| Actions | 12 weeks | | 26 weeks | |
| --- | --- | --- | --- | --- |
|  | *Control (n=369)* | *Intervention (n=336)* | *Control (n=337)* | *Intervention (n=300)* |
| Taking action to manage weight, n (%) | 247 (66·9) | 275 (81·8) | 195 (57·9) | 204 (68·0) |
| No action, n (%) | 122 (33·1) | 61 (18·2) | 142 (42·1) | 96 (32·0) |
|  |  |  |  |  |
| Actively using the ARTEMIS app, n (%) | - | 157 (46·7) | - | 80 (26·7) |
| Using learned strategies from the ARTEMIS app, but not actively using app, n (%) | - | 96 (28·6) | - | 87 (29·0) |
|  |  |  |  |  |
| Other Effective action, n (%) | 125 (33·9) | 99 (29·5) | 111 (32·9) | 76 (25·3) |
| Using another online or app-based weight loss programme | 77 (20·9) | 57 (17·0) | 70 (20·8) | 40 (13·3) |
| Attending a weight loss programme where I see someone face-to-face | 16 (4·3) | 18 (5·4) | 15 (4·5) | 14 (4·7) |
| Using weight loss medication | 24 (6·5) | 14 (4·2) | 13 (3·9) | 16 (5·3) |
| Using a meal replacement programme | 23 (6·2) | 22 (6·5) | 23 (6·8) | 14 (4·7) |
| Other (e.g., surgery) | 0 (0) | 1 (<1) | 2 (<1) | 0 (0) |
| Self-help strategies, n (%) | 117 (31·7) | 41 (12·2) | 76 (22·6) | 40 (13·3) |

**Answers were not mutually exclusive, i.e., participants could select more than one action that they used to manage their weight.*

*Table S10: Sensitivity analysis excluding those who utilised any other effective actions for weight loss.*

| Timepoint | Variable | Mean (SD) change from baseline | | | | Adjusted difference  (95% CI) | *P value* |
| --- | --- | --- | --- | --- | --- | --- | --- |
|  |  | Control | n | Intervention | n |  |  |
| 12 weeks | Weight (kg) | -1·71 (3·7) | 199 | -2·72 (4·2) | 210 | -1·19*  (-2·03 to -0·36) | 0·005 |
|  | n (%) losing ≥5% weight | 24 (12·1) | 199 | 51 (24·3) | 210 | 2·34†  (1·38 to 4·04) | <0·001 |
| 26 weeks | Weight (kg) | -1.55 (4·2) | 180 | -3·97 (6·4) | 188 | -2·40*  (-3·27 to -1·53) | <0·001 |
|  | N (%) lost ≥5% weight | 26 (14·4) | 180 | 70 (37·2) | 188 | 3·53†  (2·14 to 5·97) | <0·001 |

**Adjusted mean difference (kg) (95% CI) calculated using linear mixed effects models with fixed effects for condition, week and group*week interaction, and a random effect for participant. IMD and baseline BMI were included as covariates as baseline values were predictive of missingness in the data.*

*†Odds ratio.*

*Table S11: Codes and subcategories from content analysis for self-reported barriers to planned daily action completion.*

| Subcategory | Codes |
| --- | --- |
| Time-management & confliction priorities | Too busy or lack of time |
|  | Work commitments |
|  | Family Responsibilities |
|  | Prioritising or distracted by other tasks |
|  | Not convenient |
| Physical challenges | Illness |
|  | Physical limitations/ discomfort |
|  | Hormonal changes |
|  | Controlling blood sugar |
|  | Too tired |
| Motivational factors | Lack of motivation |
|  | Lack of willpower or self-control |
|  | Didn't keep on track |
| Dietary Challenges | Cravings |
|  | Hunger/fullness |
|  | Snacking |
|  | Comfort/pleasure eating & drinking |
|  | Cheating/taking a break from diet |
|  | Binging |
|  | Temptation |
| Social Influences | Social plans/eating out |
|  | Guests/visitors |
|  | Social expectations/pressure |
|  | Special occasions |
| Environmental Influences | Travel |
|  | On holiday |
|  | Weather |
|  | Food availability |
|  | Weekend |
|  | Environmental structure |
| Psychological Factors | Stress & mental health |
|  | Boredom |
|  | Personal life crisis |
|  | Mindfulness & mindless eating |
|  | Difficulty breaking habits |
| Planning & Organisation | Planning/preparation |
|  | Need a reminder |
|  | Forgot |
|  | Change to plans or routine |
|  | Went out or away from the house |
| Programme-related factors | Doubt about the effectiveness of action |
|  | App issues |
|  | Unsuitable or irrelevant action |
|  | Action too difficult or disliked action |
|  | Just didn’t do action |
|  | Partially performed action |
|  | Performed suitable alternative |
| Other | Don't know |
|  | Unknown/other |

*Table S12: Participant self-reported rating of heath, quality of life, and body satisfaction answered of a 5-point Likert scale.*

| **Variable** | **Self-reported rating** | **Baseline** | | **12 weeks** | | **26 weeks** | |
| --- | --- | --- | --- | --- | --- | --- | --- |
|  |  | *Control (n=806)* | *Intervention (n=800)* | *Control (n = 373)* | *Intervention (n=338)* | *Control (n=337)* | *Intervention (n=300)* |
| **Health** | Excellent, n (%) | 12 (1·5) | 7 (<1) | 4 (1·1) | 8 (2·4) | 8 (2·4) | 8 (2·7) |
|  | Very Good, n (%) | 79 (9·8) | 82 (10·3) | 57 (15·3) | 54 (16·0) | 42 (12·5) | 52 (17·3) |
|  | Good, n (%) | 311 (38·6) | 283 (35·4) | 145 (38·9) | 140 (41·4) | 133 (39·5) | 119 (39·7) |
|  | Fair, n (%) | 302 (37·4) | 339 (42·3) | 132 (35·4) | 108 (40·0) | 118 (35·0) | 93 (31) |
|  | Poor, n (%) | 102 (12·7) | 89 (11·1) | 35 (9·4) | 28 (8·3) | 36 (10·7) | 28 (9·3) |
|  | Odds ratio*  (95% CI) | - | 0·94  (0·78 to 1·12) | - | 1·19  (0·91 to 1·56) | - | 1·27  (0·96 to 1·70) |
|  | P value | - | 0·47 | - | 0·21 | - | 0·09 |
| **Quality of life** | Excellent, n (%) | 14 (1·7) | 19 (2·4) | 15 (4·0) | 14 (4·1) | 15 (4·5) | 19 (6·3) |
|  | Very Good, n (%) | 132 (16·4) | 107 (13·4) | 85 (22·7) | 83 (24·6) | 73 (21.7) | 63 (21·0) |
|  | Good, n (%) | 323 (40·1) | 307 (38·4) | 151 (40·5) | 139 (41·1) | 137 (40·7) | 128 (42·7) |
|  | Fair, n (%) | 263 (32·6) | 286 (35·8) | 100 (26·8) | 87 (25·7) | 89 (26·4) | 71 (23·7) |
|  | Poor, n (%) | 74 (9·2) | 81 (10·1) | 22 (5·9) | 15 (4·4) | 23 (6·8) | 19 (6·3) |
|  | Odds ratio*  (95% CI) | - | 0·85  (0·72 to 1·03) | - | 1·12  (0·86 to 1·47) | - | 1·13  (0·85 to 1·50) |
|  | P value | - | 0·09 | - | 0·41 | - | 0·41 |
| **Body satisfaction** | Very satisfied, n (%) | 1 (<1) | 2 (<1) | 1 (<1) | 1 (<1) | 0 (0) | 3 (1) |
|  | Satisfied, n (%) | 5 (<1) | 3 (<1) | 16 (4·3) | 19 (5·6) | 7 (2·1) | 28 (9·3) |
|  | Neither satisfied nor dissatisfied, n (%) | 23 (2·9) | 25 (3·1) | 37 (9·9) | 50 (14·8) | 44 (13·1) | 48 (16) |
|  | Dissatisfied, n (%) | 303 (37·6) | 286 (35·8) | 141 (37·8) | 165 (48·8) | 150 (44·5) | 132 (44) |
|  | Very dissatisfied, n (%) | 474 (58·8) | 484 (60·5) | 178 (47·7) | 103 (30·5) | 136 (40·4) | 89 (29·7) |
|  | Odds ratio*  (95% CI) | - | 0·94  (0·77 to 1·14) | - | 1·88  (1·42 to 2·50) | - | 1·80  (1·35 to 2·42) |
|  | P value | - | 0·52 | - | <0·001 | - | <0·001 |

** Odds ratio (95% confidence interval) calculated using ordinal logistic regression model to compare between treatment groups.*


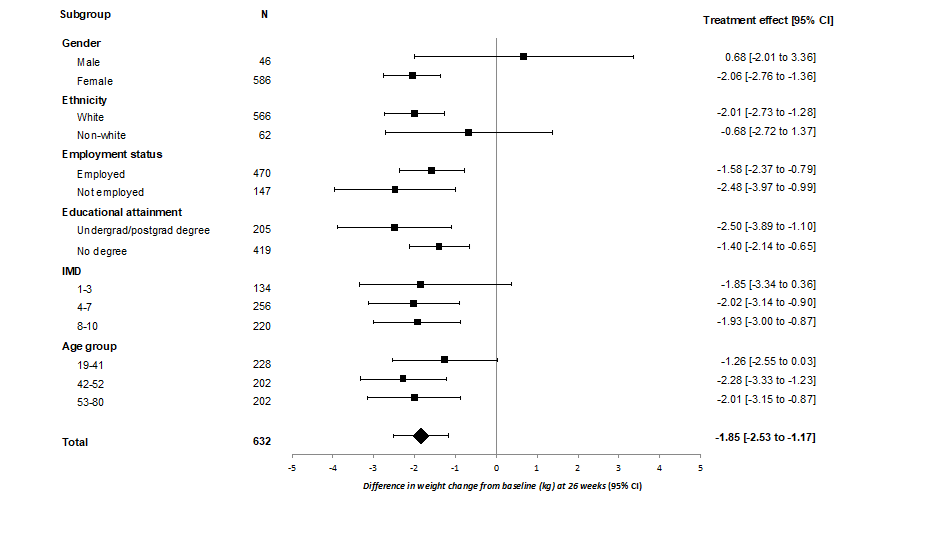


*Fig. S1: Difference in weight change from baseline to 26 weeks between intervention and control by selected sub-groups.*


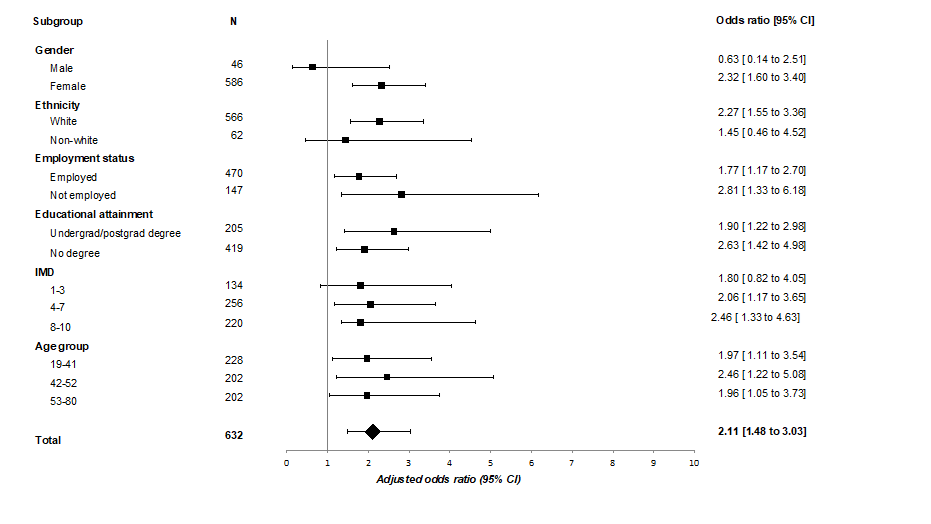


*Fig. S2: Adjusted odds ratio of losing >5% baseline body weight at 26 weeks by selected sub-groups.*
