# Supplementary material for: The effectiveness and safety of a mobile application-based self-regulation intervention to support weight loss among adults living with obesity: a large-scale pragmatic randomised controlled trial
Source: BMC Med. 2025 Nov 29;24:6. doi: 10.1186/s12916-025-04519-8 (PMC12771856; doi:10.1186/s12916-025-04519-8)
Supplement: Supplementary file 2 — Additional file 2: Overview of the weight loss action categories, individual weight loss actions, “What to do” and “Why does it matter?” descriptions, and associated tips, in the ARTEMIS mobile application. [file 12916_2025_4519_MOESM2_ESM.docx]

**Additional file 1. ARTEMIS actions**

**Overview of actions**


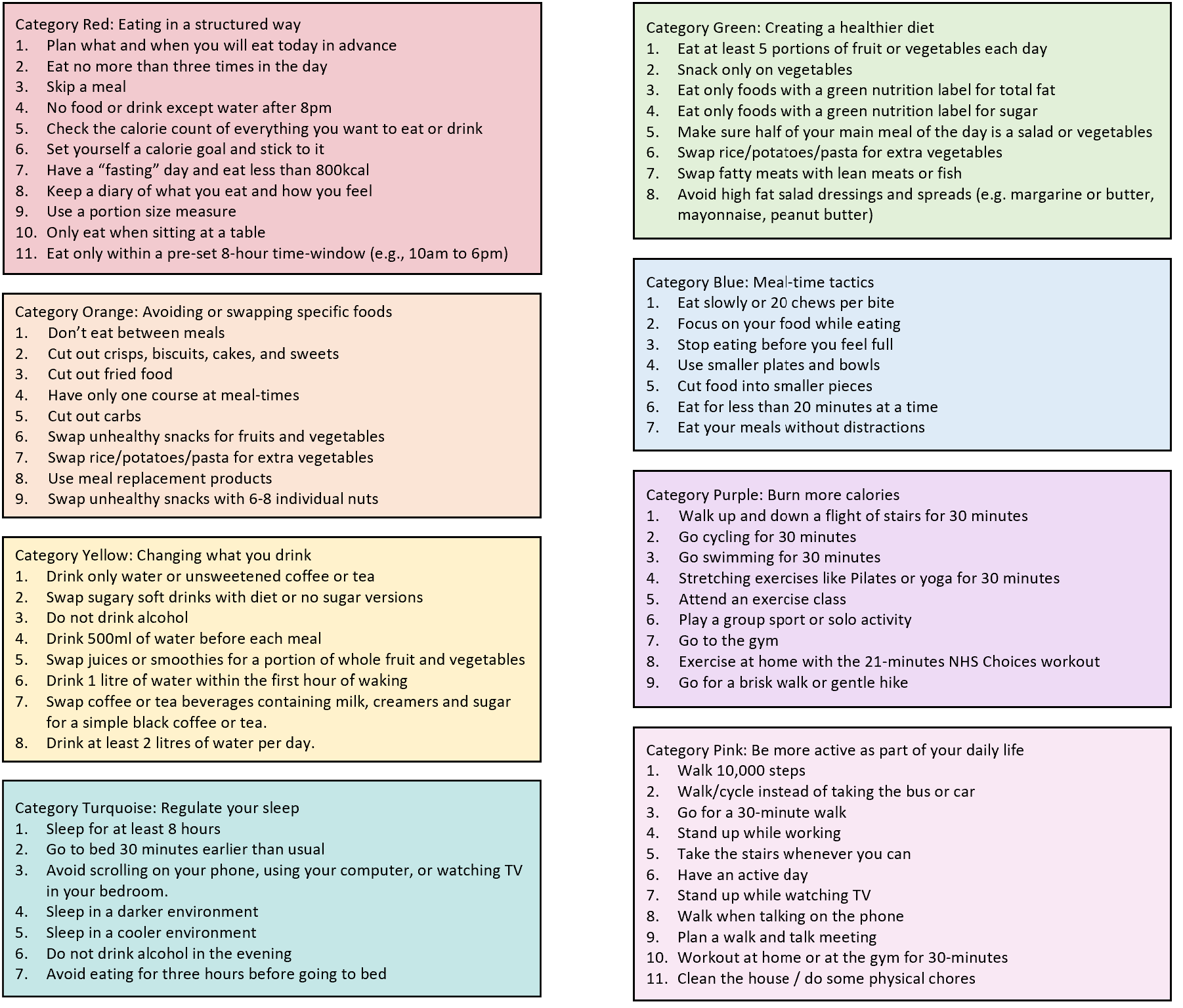


**Weight loss action category: regulate your sleep (turquoise)**

| **No** | **Action** | **What to do** | **Why does it matter?** | **Tips (see next section)** |
| --- | --- | --- | --- | --- |
| 1 | Sleep for 8 hours | Plan what time you need to wake up in the morning and try to ensure you are asleep at least 8 hours before that time. | Evidence suggests that sleep plays a role in weight loss. Insufficient sleep can increase overeating unhealthy food choices. Losing sleep may also mean you have less energy for exercise, physical activity as well as overcoming temptations. | C, H7, H8 |
| 2 | Go to bed 30 minutes earlier than usual | If you typically sleep less than 8 hours per night, try setting an alarm or other reminder to go to bed 30 minutes earlier than you normally would. If you typically get much less than 8 hours sleep you may wish to increase this. | Evidence shows there is a link between fewer total hours of reported sleep and excess bodyweight. Among a range of possible factors contributing to this being tired can lead to increased hunger, reduces appetite control and feeling of fullness, and poorer food choices. | C, H7, H8 |
| 3 | Avoid scrolling on your phone, using your computer, or watching TV, in your bedroom | Avoid using any electronic devices (e.g., phone, computer, or TV) in your bedroom before going to bed. Try to leave your devices in a separate room or away from your bedside. | Exposure to the blue light of electronic devices closely before sleep can make it harder to fall asleep and reduce the quality of your sleep. Poor sleep duration and quality can increase overeating and unhealthy food choices. | C, B11, B12, B13, H8 |
| 4 | Sleep in a darker environment | Turn off all sources of light in your bedroom and close your blinds / curtains or wear a sleep mask to bed. | Sleeping in a darker environment will reduce sleep disturbance and should help you get a better-quality sleep. Poor sleep quality increases overeating and unhealthy food choices. | C, H8 |
| 5 | Sleep in a cooler environment | A bedroom temperature between 15-20 degrees Celsius is optimal for most people’s sleep. To reduce the temperature in your room try altering your thermostat, using a fan, a lighter duvet, or opening a window. | Sleeping in a cooler room can help you to fall asleep faster and will reduce sleep disturbance. Poor sleep quality increases overeating and unhealthy food choices. | C, H8 |
| 6 | Do not drink alcohol in the evening | Do not drink any alcohol in the evening, especially not within several hours of going to bed. | Alcohol can affect the quality of your sleep and disrupts your sleep cycle. Some people may find alcohol helps them get to sleep initially, but this is outweighed by the negative effect on sleep quality through the night. Poor sleep quality increases overeating and unhealthy food choices. | A, C, D6 |
| 7 | Avoid eating for three hours before going to bed | Do not eat or drink anything (except from water) three hours before going to bed. Make sure you plan your evening meal 3 hours before going to bed and ensure you don’t have any evening snacks. | Eating or drinking too close to bedtime may impair sleep quality and is associated with increased risk of waking up. Not eating for 3 hours before bed will also help you reduce your overall calorie intake throughout the day. | A, C, D |
| 8 | Do not drink caffeine after 12pm | Stop drinking caffeinated beverages (e.g., coffee, some tea, energy drinks, or soft drinks such as coke) after 12 noon. You can swap these for decaffeinated versions in the afternoon. | Caffeine is a stimulant and can have a disruptive effect on your sleep. It will make it hard for you to fall asleep and reduce your total sleep time. It can take up to 10 hours to completely clear caffeine from your bloodstream. Evidence suggests that good quality plays a role in weight loss. | A, B1, B9, E1 |

**Weight loss action category: eating in a structured way (red)**

| **No** | **Action** | **What to do** | **Why does it matter?** | **Tips (see next section)** |
| --- | --- | --- | --- | --- |
| 1 | Plan what and when you will eat today in advance | Take the time to plan what you will eat over the next 24 hours. Make it quite detailed – when will you eat, what will you cook, will you take something prepared if you’re going out? If you know you are eating out, look up their menu in advance and plan your order. Then make sure that you stick to your plan. | A lot of calories are added to our diet from impulsive snacks or poor food choices. Committing to a food plan in the morning, when you are mindful of your goals and not exposed to temptations, can help you eat healthily throughout the day. | A, B, C, D, H |
| 2 | Eat no more than three times in the day | Make sure that you have no more than three eating occasions throughout the day. You can have breakfast, lunch, and dinner, but no snacks in between or after. | Impulsive snacking between meals can add a lot of calories. Cutting out snacks will reduce your daily energy intake. | A, B, C, D |
| 3 | Skip a meal | Skip either breakfast, lunch, or dinner. Make sure you don’t compensate for skipping the meal by snacking instead. | Skipping a meal is an easy way to reduce your overall calorie intake throughout the day. | A, B, C, D |
| 4 | No food or drink except water after 8pm | Do not eat any food after 8pm and ensure any drinks you consume after 8pm have zero or very few calories (e.g., water or black tea). | Food and drink consumed in the late evening are often unhealthy and high in calories (e.g.. crisps, chocolate, alcohol). Cutting out all foods and calorie-rich drinks in the evening can help reduce your overall calorie consumption. | A, B, C, D |
| 5 | Check the calorie count of everything you want to eat or drink | Use nutrition labels, websites, or apps to check the calorie content of food before you eat it. Make sure you consider how the calories are presented as they sometimes refer to a portion size or per 100g. Compare the calorie content with that of similar items and see if there are lower calorie alternatives. Make a conscious decision about whether you want to consume the food or drink or go for a lower calorie alternative. | Many people don’t know how many calories their foods and snacks contain. You might realise that a lower calorie alternative will be just as satisfying. | A, B, C, D, E |
| 6 | Set yourself a calorie goal and stick to it | Go to the following website to find out how many calories you need per day: <https://www.bbc.com/food/diets/how_many_calories_do_you_need>. Subtract 600 calories and set the resulting figure as your goal for the day. Then keep track of the calorie content of everything you eat throughout the day. Ensure you do not exceed your target. You can use free calorie counting mobile phone apps, such as MyFitnessPal. | Research shows that setting calorie goals can help you lose weight. It makes your goal for the day more tangible and helps you think about how to spread your daily calorie intake. | A, B, C, D, E |
| 7 | Have a “fasting” day by eating less than 800kcal | Have a day of “fasting” where you consume less than 800 calories (kcal). You can find some low calorie recipes to help you here: <https://www.bbc.com/food/collections/intermittent_dieting_recipes> <https://www.bbc.com/food/diets/low-calorie_diet>  Make sure you drink at least 2 litres of low-calorie fluids to avoid dehydration. You can use free calorie counting apps, such as MyFitnessPal, to help you keep track. | Eating only 800 calories means cutting out more than half of the calories you would consume on a normal day. | A, B, C, D, E |
| 8 | Keep a diary of what you eat and how you feel | Keep a record of all the foods and drinks you consume throughout the day. Make a note of the time when you consume them, and also the reason e.g., whether you were craving this kind of food, wanted to reward yourself, or were hungry. Write down how you felt after consuming the food or drink. Go through your food diary in the evening and see which foods or drinks you could have avoided. | Keeping a diary will make you think more about how you use food not only to feed yourself but also to provide comfort or reduce boredom. You can then think of other strategies to handle these emotions that do not include food. | C, D3 |
| 9 | Use a portion size measure | Weigh everything before you eat it and check your portion against the recommended serving size on the packet or by using this general guide: <https://blog.myfitnesspal.com/essential-guide-portion-sizes/> | Most people are unaware of recommended serving sizes. Checking portion sizes will help to prevent you eating over the recommended amount. | A, B, C |
| 10 | Only eat when sitting at a table | Only eat while you are sitting at a dining table. Don’t eat while you’re on the go or when working. | When you eat while doing other tasks, it is easy to overeat. Sitting down at a dining table will help you focus on your food and be more mindful of what and how much you’re eating. By committing to only eating while sitting at the table, you will reduce snacking. | A, B, C, D |
| 11 | Eat only within an 8-hour time window (e.g., 10am - 6pm) | Plan to eat all your meals and snacks for the day within a pre-set 8-hour time frame, for example between 10am to 6pm. Do not eat or drink anything apart from water and other no-calorie beverages, like plain coffee or tea, in the remaining 16 hours of the day. | Restricting your eating window to 8 hours per day can cut your calorie intake over the course of the day, which in turn may contribute to weight loss. | A, B, C, D, H7 |

**Weight loss action category: avoiding or swapping specific foods (orange)**

| **No** | **Action** | **What to do** | **Why does it matter?** | **Tips (see next section)** |
| --- | --- | --- | --- | --- |
| 1 | Do not eat between meals | Do not eat any snacks between meals. Stick to your three main meals, breakfast, lunch and dinner. | Impulsive snacking between meals can add lots of calories to your overall daily intake. Cutting out snacks between meals will reduce the overall amount you consume in day. | A, B, C, D |
| 2 | Cut out crisps, biscuits, cakes, and sweets | Do not eat any crisps, biscuits, or highly processed baked goods (e.g., pastries and croissants), cakes, chocolates, and sweets throughout the day. | These foods are highly ‘energy dense’ and contain a lot of calories in each bite, without being nutritious. You would have to eat a lot to fill you up so it’s easy to overeat them. Cutting them out will reduce your calorie intake. | A, B, C, D |
| 3 | Cut out fried foods | Do not consume any fried foods today. This includes for example fries or chips, onion rings, poppadoms, battered fish. | Fried foods are highly ‘energy dense’ and contain a lot of empty calories, without being nutritious. You would have to eat a lot to fill you up so it’s easy to overeat them. Cutting them out will reduce your calorie intake. | A, B, C, D, E |
| 4 | Have only one course at meal-time | Do not consume any starters or desserts with your lunch and dinner. For breakfast, consume just one type of food (e.g., only toast or cereal). | Starters and desserts add to your calorie intake. Cutting them out will reduce your consumed calories. | A, B, C, D |
| 5 | Cut out carbohydrates | Avoid carbohydrates, including potatoes, rice, pasta, bread, breakfast cereals, beans, and sugary foods such as pastries, cakes, biscuits, confectionery and chocolates. Meat, fish, poultry, fruit / green vegetables, and dairy are allowed. | Carbohydrates account for around 40% of all the calories we eat. When you cut out carbohydrates, you will tend to reduce your overall energy intake. | A, B, C, D, E |
| 6 | Swap unhealthy snacks for fruits and vegetables | Swap unhealthy snacks with fruits and vegetables, such as apples, carrots, celery, or peppers. | Unhealthy snacks are high in calories, but usually low in calories. Swapping them with fruits and vegetables will reduce your calorie intake. | A, B, C, D |
| 7 | Swap rice, potatoes, and pasta for extra vegetables | Avoid rice, potatoes, and pasta as a side to your main course. Instead swap them with boiled or steamed vegetables, including broccoli, carrots, or cabbage. | Rice, potatoes and pasta contain far more calories than vegetables. Replacing starchy carbohydrates with vegetables will help you stay full while eating fewer calories. | C, D, E |
| 8 | Use meal replacement products | Try swapping breakfast, lunch and/or dinner for a meal replacement product such as a specially formulated meal bar, shake, or soup. You can buy these online or in your local pharmacy. | Specially formulated bars, shakes, or soup shakes contain all the nutrients you need and are usually much lower in calories than a typical meal. | A, B, C, D |
| 9 | Swap unhealthy snacks with 6-8 individual nuts | Replace all unhealthy snacks with 6-8 nuts. | Nuts are high in protein and fibre which will help you to feel fuller for longer. | A, B, C, D |

**Weight loss action category: changing what you drink (yellow)**

| **No** | **Action** | **What to do** | **Why does it matter?** | **Tips (see next section)** |
| --- | --- | --- | --- | --- |
| 1 | Drink only water or unsweetened coffee or tea | Drink only water, coffee and tea today. Your tea or coffee may include a small amount of milk, but no sugar, honey, or syrups. | High calorie drinks can quickly increase your calorie intake without increasing your sense of fulness. Switching to low-calorie drinks will help you lose weight. | C, D4, E1 |
| 2 | Swap sugary soft drinks with diet or no sugar versions | Swap your sugary soft drinks for the zero or low-sugar versions. This includes sodas, sport drinks, and energy drinks. You might have to experiment with different brands to discover your preferred taste. | A typical can of sugary drink contains about 100 calories. Switching to zero or low-sugar versions of soft drinks can help reduce your calorie intake. | C |
| 3 | Do not drink alcohol | Refrain from drinking any alcohol today. | Alcohol contains a lot of calories. A pint of beer contains about 250 calories, 150ml of red wine contain 125 calories. By avoiding drinking alcohol, you can reduce the calories you consume. | C, D6 |
| 4 | Drink 500ml of water before each meal | Drink 500ml of water before you choose your meal and decide on the portion size. | Drinking 500ml of water can help fill up your stomach. That way you will feel less hungry when making meal decisions, helping you to make healthier choices and choose smaller portion sizes. | C, E1 |
| 5 | Swap juices or smoothies for a portion of whole fruit and vegetables | Avoid juices or smoothies and eat a piece of whole fruit or vegetable instead. | Juices and smoothies tend to be highly processed with a high calorie and sugar content. Swapping juice with whole fruits and vegetables has the advantage that you benefit from the fibre they contain. Fibre is good for digestion, cholesterol, and helps you feel fuller for longer. Eating whole fruits and vegetables rather than drinking a smoothie slows down the rate of eating and increases feelings of fullness. | C |
| 6 | Swap coffee or tea containing milk, creamers, and sugar for a black coffee or tea | Swap high-calorie coffee or tea beverages containing milk, creamers, sugar or syrups for a black coffee or tea. | Switching to black coffee or tea without milk, creamers, sugar, or syrups will help you reduce your calorie intake. | A, C, D4 |
| 7 | Drink at least 2 litres of water per day | Drink at least 2 litres of water today. | Drinking 2 litres of water per day can help fill up your stomach. That way you will feel less hungry when making meal decisions, helping you to make healthier choices, and choose smaller portion sizes. | C, E1 |

**Weight loss action category: creating a healthier diet (green)**

| **No** | **Action** | **What to do** | **Why does it matter?** | **Tips (see next section)** |
| --- | --- | --- | --- | --- |
| 1 | Eat at least 5 portions of fruit or vegetables each day | Eat at least five portions of different fruits and vegetables. | Fruits and vegetables typically contain few calories and are nutrient dense, so can help bulk out a meal. This will help you to feel fuller on fewer calories. | C, H |
| 2 | Snack only on vegetables | Tempted to snack outside of your three main meals? Then snack on vegetables, such as carrots, peppers, or cucumber. | Vegetables are low in calories and consuming them as a snack won’t add too many calories to your daily intake. | A, B, C, D |
| 3 | Eat only foods with a green nutrition label for total fat | Only eat foods with low total fat (3g or less per 100g). This is often indicated by a green colour-coding for total fat on the nutrition label. | Fat contains a lot of calories. Eating low-fat foods will therefore reduce your calorie intake. | A, B, C, D, E |
| 4 | Eat only foods with a green nutrition label for sugar | Only eat foods with low sugar content (5g or less per 100g). This is often indicated by a green colour-coding for sugars on the nutrition label. | Sugary foods contain a lot of calories. Eating low-sugar foods will reduce your calorie intake. | A, B, C, D, E |
| 5 | Make sure half of your main meal for the day is a salad or vegetables | Make sure that half of your main meal of the day - lunch or dinner - consists of boiled or steamed vegetables, or a salad. Potatoes do not count as vegetables. Salad dressings should be low fat, such as lemon juice, balsamic vinegar, or yoghurt dressing. | Vegetables provide you with many important nutrients and are low in calories. Salads, and steamed or boiled vegetables, are a great side to your main meal. They add bulk to a meal so you feel fuller and satisfied. | C, D, E |
| 6 | Swap rice,  potatoes, and pasta with extra vegetables | Avoid rice, potatoes, and pasta. Instead swap for boiled or steamed vegetables, including greens, carrots, or parsnips. | Rice, potatoes and pasta contain far more calories than vegetables. Replacing starchy carbohydrates with vegetables will help you stay full while eating fewer calories. | C, D, E |
| 7 | Swap fatty meats with lean meats or fish | Avoid fatty meats, including salamis, sausages, steaks, pork belly, or high fat minces. Swap them with lean cuts of meat which have a relatively low-fat content. Remove all visible fat from meat, including the skin from chicken. Choose extra lean mince or ask your butcher for a leaner cut. Or choose fish instead. | Fatty meats contain many calories. You can reduce your calorie intake by switching to lean meats. | B, C, D |
| 8 | Avoid high fat salad dressings and spreads | Avoid high fat salad dressings and spreads such as margarines, mayonnaise, butter and peanut butter. | High fat salad dressings and spreads contain many calories. One tablespoon of mayonnaise or butter contains about 100 calories. Avoiding eating high fat salad dressings and spreads will reduce your calorie intake. | A, B, E |

**Weight loss action category: meal-time tactics (blue)**

| **No** | **Action** | **What to do** | **Why does it matter?** | **Tips (see next section)** |
| --- | --- | --- | --- | --- |
| 1 | Eat slowly (e.g., 20 chews per bite) | Slow down how quickly you eat. You can achieve this by chewing each bite twenty times, decreasing your chewing speed, or putting your cutlery down between bites. | Reducing your eating speed will help you notice feelings of fullness before you have overeaten. | C, H |
| 2 | Avoid distraction and focus on your food while eating | Eat your meals without distractions e.g., do not use your phone, watch TV, or read a book whilst eating. Be mindful about eating, and your feelings of satisfaction and fullness. | Being mindful can help you identify feelings of fullness and support you in avoiding overeating. | C, D |
| 3 | Stop eating before you feel full | Stop eating before you feel full. Instead look out for the moment when you stop feeling hungry, and stop then. You can freeze leftovers or keep them in the fridge for another time. | It takes a while for feelings of fullness to set in.  Stopping eating at the moment you don’t feel hungry anymore, will prevent you overeating. | B, C, D |
| 4 | Use smaller plates and bowls | Use smaller plates or bowls and smaller serving spoons to help with your portion control. | Using smaller crockery and utensils will help you eat smaller portions and reduce your calorie intake. | C, D |
| 5 | Cut food into smaller pieces | Cut your food into smaller pieces when eating. | Reducing the bite size will increase the time you need to eat. This will provide your gut hormones more time to tell your brain you are full, meaning you can feel satisfied before you have overeaten. It will also give you the feeling of having had a larger meal. | C, H |
| 6 | Eat for less than 20 minutes at a time | Don’t spend more than 20 minutes eating each meal. Eat at a normal pace. You can freeze your leftovers or keep them in the fridge. | Restricting the time you spend consuming your food will automatically restrict your calorie consumption. | B, C |

**Weight loss action category: burn more calories (purple)**

| **No** | **Action** | **What to do** | **Why does it matter?** | **Tips (see next section)** |
| --- | --- | --- | --- | --- |
| 1 | Walk up and down a flight of stairs for 30 minutes | Walk up and down a flight of stairs for approximately 30 minutes. If you can’t manage 30 continuously you can take a short at the bottom of the stairs and then go again. | Engaging in exercise burns calories and helps you lose body fat. 30 minutes of moderate-to-vigorous physical activity is the daily recommendation for adults. | C, F |
| 2 | Go cycling for 30 minutes | Go cycling outdoors or at the gym for 30 minutes. Take breaks if necessary. | Engaging in exercise burns calories and helps you lose body fat. 30 minutes of moderate-to-vigorous physical activity is the daily recommendation for adults | C, F, G |
| 3 | Go swimming for 30 minutes | Go swimming for 30 minutes. If you can’t manage 30 minutes continuously take a short break at the side of the pool and then go again. | Engaging in exercise burns calories and helps you lose body fat. 30 minutes of moderate-to-vigorous physical activity is the daily recommendation for adults. | C, F, G |
| 4 | Perform stretching exercises for 30 minutes (e.g., Pilates or yoga) | Do stretching exercises (e.g., Pilates or yoga) at home for 30 minutes. There are many online tutorials available, such as this one: <https://www.youtube.com/watch?v=9jAyRP0bqKA> | Stretching exercises help develop flexibility. This may enable you to engage in more physical activities. Any extra exercise helps to burn more calories. | C, F |
| 5 | Attend an exercise class | Attend a structured group exercise or sports class. This may be a class at a gym or sports club led by a trainer, e.g., a spin class, a dance class, or you could use online videos/exercise apps. | Engaging in exercise burns calories and helps you lose body fat. Doing exercise in a group can be especially motivating and fun. | C, F |
| 6 | Play a group sport or perform a solo activity | Play a group sport such as football, basketball, badminton or tennis, or a solo activity such as golf. This might either be formal training or a game between friends. | Engaging in exercise burns calories and helps you lose body fat. Doing exercise in a group can be especially motivating and fun. | C, F, G |
| 7 | Go to the gym | Go to the gym for a workout. A good way to structure your training is to start with a warm-up on a cardio machine, followed by strength training, and a light cardiovascular cool-down exercise to finish. Make sure to ask the trainers or someone else at the gym to explain any unfamiliar equipment. Most equipment will also have useful diagrams and instructions. | The suggested gym routine will help you strengthen several muscles in your body, which helps boost your long-term metabolic rate. The cardio routine will get your heart rate going and burn calories. | C, F, G, H6, H7 |
| 8 | Exercise at home with the 21-minutes NHS Choices workout | Try one of the NHS Choices website 21-minute workouts that you can perform easily at home. They include: a 6-minute warm-up, 10-minute workout of your choice and 5-minute cool-down. You can find them here: <https://www.nhs.uk/live-well/exercise/10-minute-workouts/> | The NHS Choices workouts will strengthen several body muscles. Building muscle mass will help boost your long- term metabolic rate. Plus, engaging in exercise burns calories. | C, F |
| 9 | Go for a brisk walk or gentle hike | Go for a brisk walk or gentle hike until you are out of breath and can no longer sustain a conversation. | Engaging in exercise burns calories and helps you lose body fat. | C, F, G |

**Weight loss action category: be more active as part of your daily life (pink)**

| **No** | **Action** | **What to do** | **Why does it matter?** | **Tips (see next section)** |
| --- | --- | --- | --- | --- |
| 1 | Walk 10,000 steps | Set yourself the goal of walking at least 10,000 steps. You can count your steps using a pedometer or fitness tracker. There are many free apps that have pedometer functionality as well. They record the number of steps you made on your phone. | Walking 10,000 steps in one day burns around 500 calories, which helps you lose body fat. | C, F |
| 2 | Walk or cycle instead of taking the bus, train, or car | If you are going somewhere, cycle or walk some or all of the way. | Engaging in physical activity burns calories and helps you lose body fat. | C, F |
| 3 | Go for a 30-minute walk | Go for a 30-minute walk instead of sitting down, this can either be with others or alone. | You burn more calories while walking than sitting. Any extra physical activity helps to burn more calories. | C, F, G |
| 4 | Stand up while working | Stand up while you’re working. If your work place does not have a height adjustable desk, try to find a cupboard or cabinet that has a good height to work on. Alternatively, try to have standing or walking meetings with your colleagues if more practical. | You burn more calories standing than sitting. | C, F |
| 5 | Take the stairs whenever you can | Always choose the stairs over the lift. | Engaging in physical activity burns calories and helps you lose body fat. | C, F |
| 6 | Have an active day | Have an active day, for example go for a hike or play a sport such as football, golf, or tennis. This can either be with friends or family, or alone. | Engaging in physical activity burns calories and helps you lose body fat. Doing this together with family or friends can make it more enjoyable. | C, F, G |
| 7 | Stand up while watching TV | Stand up while watching TV. | You burn more calories standing than sitting. Any extra physical activity helps to burn more calories. | C, F |
| 8 | Walk when talking on the phone | Every time you are talking on the phone, get up and walk around. Do this for the full duration of the phone call. | You burn more calories while walking than sitting. Any extra physical activity helps to burn more calories. | C, F |
| 9 | Plan a walk-and-talk meeting with friends or colleagues | Plan a meeting with friends, or one of your work meetings to done whilst walking. | You burn more calories while walking than sitting. Any extra physical activity helps to burn more calories. | C, F, G |
| 10 | Fit mini-workouts into your daily routine | Do some stretching exercises when you get out of bed in the morning, squats, lunges or push ups before you get in the shower, run up flights of stairs throughout your day, walk at a fast pace pushing hard off your toes with each step, or bicep curl your shopping bags while walking. | Any extra physical activity helps to burn more calories. | C, F, H7 |
| 11 | Do some physical chores around the house | Do some physically demanding chores today such as mowing the lawn, cleaning the windows, rearranging the furniture, cleaning out the garage, or brushing / hoovering the floors. | Any extra physical activity in your day helps to burn more calories. | C, F |

**Tips and Tricks: What to do to succeed**

If you are experiencing difficulties completing your planned action today, have a look at the tips below *(check the right-side column of the action plan list to discover which tips are relevant).*

**B) Dealing with temptation**

1. Avoid places with food and drink temptations, such as fast-food restaurants, ice cream parlours, or coffee shops.
2. Do not go shopping when you are hungry.
3. Make a shopping list and stick to it.
4. If you have food or drinks in your house that aren’t part of your eating plan, give them to a local food bank or your friends/neighbours.
5. Don’t bring unhealthy foods/drinks into the house.
6. If you’re having an unhealthy snack (e.g., a piece of chocolate), take only a small amount and return the rest to the fridge/cupboard.
7. Make sure you have healthy snacks at home and a bottle of water with you when you’re out.
8. When you are going to eat out, check the menu beforehand and choose what you will eat. Restaurants are required by law to provide nutritional information about their dishes, including calorie content. If the information is not included on the menu, ask the waiter. In chain restaurants the nutritional information might also be available online.
9. If you tend to reward yourself by eating food or having certain drinks, try to find a reward in something else, e.g., going to the cinema, or taking a hot bath.
10. If you think you might struggle to find healthy foods in your lunch break, prepare your lunch at home and bring it with you to work.
11. Turn off distracting notifications on your phone
12. Try charging your phone at the other side of your bedroom, or in another room, or putting it away at least half an hour before bed.
13. Create a night-time routine that doesn’t include your phone, computer, or television such as going straight to sleep, reading a book, or journaling.

**A) Dealing with cravings**

1. Make a bargain with yourself to wait a minute to see if the craving passes. Then see if you can last another day.
2. Eat chewing gum or brush your teeth instead. You might not feel like chocolate after that!
3. Distract yourself e.g., by walking up and down the stairs or phoning a friend.
4. Think about how it will be once you have lost weight. Really try to imagine what it will feel like.
5. Acknowledge the feeling of craving, think about how it feels. Don’t wrestle with it and don’t stress about it. You don’t need to worry about what it means or what will happen, just notice the feeling. Be mindful.
6. Drink a glass of water. If you dislike the taste of plain water, try infusing it with lemon, cucumber, ginger, or mint leaves.

**D) Staying strong despite social influences**

1. Learn to say “no”. Practice saying “thanks, but no” until it becomes a habit.
2. If you feel uncomfortable using a written food diary in public, make notes on your phone. Or take a photo and write it down later.
3. Explain to family and friends why losing weight is important to you and ask for their support.
4. Ask those around you to keep unhealthy foods and drinks out of your sight.
5. If you are attending a social gathering, make sure you bring yourself some healthy food options.
6. If people around you are drinking alcohol choose a low-calorie beverage instead e.g., spirit and diet mixer.

**C) Dealing with drops in motivation**

1. Imagine how you will feel once you have lost weight: What will you look like? What will you be able to do? What will it mean to you?
2. Plan a (non-food) reward for yourself once you have lost a certain amount of weight.
3. Write down a list of reasons why you want to lose weight and stick it on your wall.
4. Stick a photo on your wall from a time when you were happy with your weight.
5. Review your progress so far in the weight tracking app. What does it mean to you? Where would you like to see the graph go?
6. Go through your wardrobe and find clothes that currently don’t fit. Imagine wearing them again.

**E) If you’re not sure about the food and drink alternatives**

1. If you struggle drinking plain water, try infusing it. Perhaps with slices of lemon, orange, cucumber, ginger, or mint leaves. Or try sparkling water.
2. If you’re not a fan of cooked/boiled vegetables, try roasting them or eating them raw.
3. If you don’t like green vegetables, try mashing them together with other vegetables to create a combined flavour.
4. If you dislike the taste of boiled or steamed vegetables, add lemon juice, herbs, or spices.

**H) Time, cost, and organisation issues**

1. Prepare your meals in advance as this will be cheaper than eating out.
2. Look out for special offers on fruits and vegetables.
3. Prepare meals in bulk and put them in the fridge or freeze them for when you don’t have much time.
4. Have healthy ready meals in the freezer for when time is short.
5. Buy healthy prepared foods such as a green salad or a lean soup for when you’re busy.
6. If gym membership is too expensive, find things to do without a gym, such as going for a run or following an exercise video on YouTube.
7. Plan in advance. Put your weekly schedule in your calendar including wake-up times, bed times, meal times, and exercise times.
8. Keep a record for what’s working for you and what’s not.

**G) Dealing with bad weather**

1. Buy appropriate clothes for the weather e.g., a waterproof jacket and trousers, or comfortable t-shirt and shorts.
2. Check the weather forecast. If the weather is going to improve, move your action to a later time of the day.
3. Find an indoor exercise. For instance, if you planned to go for a run, go running on a treadmill instead.

**F) Dealing with barriers to exercise**

1. If you are feeling stiff or sore, try exercising a different part of the body. Also try stretching the sore bits or taking a hot shower and you will feel better. Fitness exercises that are not primarily focussed on building muscles, such as walking, swimming, cycling or jogging, can help muscle soreness go away.
2. Find an exercise buddy. Having someone to exercise with is more fun and you won’t want to let them down by not showing up.
3. If you find exercising boring, listen to music, or watch television while working out.
4. If you feel uncomfortable exercising around people you don’t know, choose a time when it’s less crowded, go with a friend, or exercise at home.
