# Supplementary material for: The effectiveness and safety of a mobile application-based self-regulation intervention to support weight loss among adults living with obesity: a large-scale pragmatic randomised controlled trial
Source: BMC Med. 2025 Nov 29;24:6. doi: 10.1186/s12916-025-04519-8 (PMC12771856; doi:10.1186/s12916-025-04519-8)
Supplement: Supplementary file 3 [file 12916_2025_4519_MOESM4_ESM.docx]

Additional file 4. Electronic Case Report Forms (Screening, Baseline and Follow-up assessments)

Screening

The following text will be presented to all participants within the REDCap eCRF interface. Note. All participant facing text is non-italicised:

Screening

Please complete the survey below.

Thank you!

To determine your eligibility for this study, please answer the following 17 questions about yourself. This will take 1-2 minutes.

Date of birth (dd/mm/yyyy): (dropdown box) (18 or above=eligible; below 18=ineligible)

Sex: (Dropdown box with three options: male, female, prefer not to say)

Ethnicity: (Dropdown box with five possible options: i.e., Asian or Asian British (e.g., Indian, Pakistani, Bangladeshi, Chinese, any other Asian background); Black, Black British, Caribbean or African (e.g., Caribbean, African, any other Black, Black British, or Caribbean background); Mixed or multiple ethnic groups (e.g., White and Black Caribbean, White and Black African, White and Asian, any other mixed or multiple ethnic background); White (English, Welsh, Scottish, Northern Irish (or British), Irish, Gypsy or Irish Traveller, Roma, any other White background); Other ethnic group (Arab, any other ethnic group); prefer not to say.

Height (Feet / Inches, Metres, cms)): (dropdown box)

Weight (Stone / Pounds, Kilograms)): (dropdown box)

The answers to the two previous questions will be used to calculate BMI (weight (kg)/height (m)2). If ethnicity has been answered “White (English, Welsh, Scottish, Northern Irish (or British), Irish, Gypsy or Irish Traveller, Roma, any other White background)”, the BMI variable must be ≥ 30 to be eligible; ≥ 27.5 for all other ethnic groups.

What was the highest level of formal education you have achieved? (Dropdown box with five options: No formal education;

Some education (no qualification), GCSE/O-level (or equivalent); A level / BTEC (or equivalent); undergraduate or postgraduate degree; prefer not to say.

Are you currently living in the UK? (Yes/No) (Yes=eligible; no=ineligible)

Do you have access to the internet via a smartphone or tablet? (Yes/No) (Yes=eligible; no=ineligible)

Do you have access to a digital weighing scales? (Yes/No) (Yes=eligible; no=ineligible)

Have you attended a weight management programme in the past three months? (Yes/No) (Yes=ineligible; no=eligible)

Are you currently taking part in any other weight management study? (Yes/No) (Yes=ineligible; no=eligible)

Have you lost more than 5kg (10 pounds) body weight in the previous six months? (Yes/No) (Yes=ineligible; no=eligible)

Have you ever undergone bariatric surgery, or are you presently scheduled to have bariatric surgery? (Yes/No) (Yes=ineligible; no=eligible)

Are you pregnant, or planning a pregnancy in the next 12 months? (Yes/No) (Yes=ineligible; no=eligible)

Have you ever been diagnosed with an eating disorder? (Yes/No) (Yes=ineligible; no=eligible)

Have you recently been diagnosed with a disease, or expected to undergo treatment for a disease, associated with substantial weight loss e.g., cancer treatment? (Yes/No) (Yes=ineligible; no=eligible)

If ineligible:

We’re sorry, based on the answers you have provided you are not eligible for this study. If there has been an error, please refresh this page, or contact us on [artemis@phc.ox.ac.uk](mailto:artemis@phc.ox.ac.uk).

If eligible:

Based on the answers you have provided we can confirm you are eligible to take part in the study. Please click here to continue to the remainder of the assessment. This should take less than 5 minutes.

Baseline

The following text will be presented to all participants within the REDCap eCRF interface. Note. All participant facing text is non-italicised:

Baseline Assessment

Please complete the survey below.

Thank you!

Socio-demographic information:

Please enter your information into the spaces provided:

Title: (Dropdown box with four options: Mr. Ms, Mrs, Dr)

First name: (Free text)

Last name: (Free text)

Email address: (Free text)

Mobile number: (Free text)

Postcode: (Free text)

Current employment status (please select all that apply: (Dropdown box with nine options, with the possibility to select multiple options: Retired, employed, self-employed, long-term sick or disabled, looking after home or family, in education or training, unemployed, other (please specify))

Anthropometric measurements:

Please weigh yourself on a digital weighing scale and report your exact weight below. Ideally this should be done within an hour of waking up, on an empty stomach, having consumed no food or liquid in the previous six hours, and having refrained from vigorous physical activity for the same period of time. Please take a photograph of your weight displayed on the digital scales by clicking ‘upload file’, ‘chose file’, and then ‘Take a photo’, or you can choose a photo already in your library, and press submit. Note. Important that participants cannot progress until this is completed.

These next questions are about how you feel about yourself, your weight, your food, you’re your physical activity. 

On how many of the past seven days…

Has thinking about food, eating or calories made it very difficult to concentrate on things you are interested in (such as working, following a conversation or reading)? (0 days; 1-2 days; 3-5 days; 6-7 days)

Has thinking about your weight or shape made it very difficult to concentrate on things you are interested in (such as working, following a conversation or reading)? (0 days; 1-2 days; 3-5 days; 6-7 days)

Have you had a definite fear that you might gain weight? (0 days; 1-2 days; 3-5 days; 6-7 days)

Have you tried to control your weight or shape by making yourself sick (vomit) or taking laxatives? (0 days; 1-2 days; 3-5 days; 6-7 days)

Have you exercised in a compulsive way as a means of controlling your weight, shape or body fat, or to burn off calories? (0 days; 1-2 days; 3-5 days; 6-7 days)

Have you had a sense of having lost control over your eating (at the time that you were eating)? (0 days; 1-2 days; 3-5 days; 6-7 days)

In general, would you say your health is … (Excellent, Very good, Good, Fair, Poor.)

In general, would you say your quality of life is … (Excellent, Very good, Good, Fair, Poor.)

How satisfied are you with your body at this moment? (Very satisfied, Satisfied, Neither satisfied nor dissatisfied, Dissatisfied, Very dissatisfied)

You have now finished the baseline assessments. Please click next where we will tell you whether you get to use the app or will try to lose weight by yourself.

Follow-up

The following text will be presented to all participants within the REDCap eCRF interface at assessment timepoints at 12 and 26 weeks. Note. All participant facing text is non-italicised:

Follow-up assessment (12 weeks)

Please complete the survey below.

Thank you!

Anthropometric measurements:

Please weigh yourself on a digital weighing scale and report your exact weight into the text box provided. Ideally this should be done within an hour of waking up, on an empty stomach, having consumed no food or liquid in the previous six hours, and having refrained from vigorous physical activity for the same period of time. Where possible please use the same scale as at your previous weight in. 

Please take a photograph of your weight displayed on the digital scales by clicking ‘upload file’. Then ‘choose file’, and then ‘Take a photo’ or you can choose a photo already in your library, and press submit. Alternatively, you can also send your photo to artemis@phc.ox.ac.uk at a later time. This will not effect your reimbursement

Eating Disorders Evaluation Questionnaire Short form (modified six-item questionnaire).

We are now going to ask you some questions about disordered weight management behaviours. It is important for us to collect this information as we will want to monitor any changes in these throughout the study for your safety.

On how many of the past seven days…

Has thinking about food, eating or calories made it very difficult to concentrate on things you are interested in (such as working, following a conversation or reading)? (0 days; 1-2 days; 3-5 days; 6-7 days)

Has thinking about your weight or shape made it very difficult to concentrate on things you are interested in (such as working, following a conversation or reading)? (0 days; 1-2 days; 3-5 days; 6-7 days)

Have you had a definite fear that you might gain weight? (0 days; 1-2 days; 3-5 days; 6-7 days)

Have you tried to control your weight or shape by making yourself sick (vomit) or taking laxatives? (0 days; 1-2 days; 3-5 days; 6-7 days)

Have you exercised in a compulsive way as a means of controlling your weight, shape or body fat, or to burn off calories? (0 days; 1-2 days; 3-5 days; 6-7 days)

Have you had a sense of having lost control over your eating (at the time that you were eating)? (0 days; 1-2 days; 3-5 days; 6-7 days)

Weight management questionnaire

- Have you taken any actions to manage your weight since the last assessment? (Yes / No)

If yes:

- What have you being doing to manage your weight (please click all that apply): (participants should be able to click on multiple options from the following drop box of options: Actively using the ARTEMIS app; using learned strategies from the ARTEMIS app, but not actively using app; using another online or app based weight loss programme; attending a weight loss programme where I see someone face-to-face; using weight loss medication; using a meal replacement programme; other (please specify further in the text box below). If participant choses other options a text box should be available to provide further detail.

Finally, we are going to ask you three questions about how your wellbeing.  

In general, would you say your health is … Excellent, Very good, Good, Fair, Poor.

In general, would you say your quality of life is … Excellent, Very good, Good, Fair, Poor.

How satisfied are you with your body at this moment? Very satisfied, satisfied, neither satisfied nor dissatisfied, dissatisfied, Very dissatisfied.

Thank you, you have now finished the (12, 26) week follow-up assessments. You will receive an email shortly with confirmation our your (£8, £20) reimbursement. 

Randomised to the control group:

If 12-week assessment:

We will contact you again in advance of the next follow-up in 14-weeks to input your weight and upload your photo verification again, at which time you will receive a further (£20). We look forward to seeing you then and appreciate your continued support with this study.

All the best.

The ARTEMIS study team

Department of Primary Care Health Sciences

University of Oxford

If 26-week assessment:

As this was the final assessment timepoint, your participation in the study is now complete. Once again, we at the ARTEMIS study team would like to thank you for your contributions to this research study. We will be in contact on the email address provided to let you know the results of the study in due course.

All the best.

The ARTEMIS study team

Department of Primary Care Health Sciences

University of Oxford

Randomised to the intervention group:

If 12-week assessment:

We will also contact you again in advance of the next follow-up assessment in 14-weeks to input your weight and upload your photo verification again, at which time you will receive a further £20. We look forward to seeing you again tomorrow in the ARTEMIS app (if you are still engaging with the active intervention phase). Thank you, we greatly appreciate your continued support with this research.

All the best.

The ARTEMIS study team

Department of Primary Care Health Sciences

University of Oxford

If 26 week assessment:

Thank you for completing this assessment. This completes your involvement in the study. We will email you the results of the study when we have analysed the results from everyone. 

All the best.

The ARTEMIS study team

Department of Primary Care Health Sciences

University of Oxford
